# Replication stress primes a trophectoderm fate in embryonic stem cells

**DOI:** 10.1038/s41420-026-03169-w

**Published:** 2026-06-02

**Authors:** Andrea Gnocchi, Christelle El Kai, Cristina Castellan, Lucia Santorelli, Negar Arghavanifard, Angela Bachi, Vincenzo Costanzo

**Affiliations:** 1 Oncology and Hemato-Oncology Department, University of Milan, Milan, Italy; 2 IFOM ETS - The AIRC Institute of Molecular Oncology, Milan, Italy

**Keywords:** Stem-cell differentiation, Embryonic stem cells, Stalled forks

## Abstract

Early embryogenesis features rapid cell cycles that impose substantial replication stress (RS). How RS shapes lineage potential remains unclear. Using mouse embryonic stem cells (ESC) as an inner cell mass model, we show that RS rapidly and durably induces trophoectoderm (TE) regulators, including Cdx2, Gata3, Eomes, Elf5, Hand1 and Tfap2c. This program requires ATR checkpoint signaling as ATR inhibition suppresses RS-induced TE gene expression without restoring pluripotency markers, indicating an active, checkpoint-driven fate response rather than passive identity loss. Additionally, RS activates JNK, increasing c-JUN abundance and phosphorylation. Blocking JNK attenuates TE induction, placing AP-1 as a cooperating pathway. The TE bias persists after stress withdrawal, promoting acquisition of a TE-like phenotype and enhancing ESC contribution to extraembryonic compartments in embryo-like structures. Quantitative chromatin proteomics reveals eviction of nucleosomes and condensin along with enrichment of DNA repair factors and ATR cofactors, consistent with a chromatin-competent and checkpoint-responsive state, permissive for lineage rewiring. These findings define an ATR- and JNK/c-JUN-dependent stress response axis that redirects embryonic cell identity toward TE, linking RS to developmental plasticity. If reactivated in the adult, this pathway could lead to somatic fate shifts that may predispose to malignancy.

## Introduction

Mouse embryonic stem cells (ESC) are known for their ability to generate all embryonic lineages [1, 2]. However, differently from the totipotent zygote, ESC are not able to give rise to extraembryonic tissues, such as the trophectoderm (TE) [3, 4]. In addition, when injected in developing morulae, ESC typically participate in the development of the embryo proper, but cannot be found in the extraembryonic compartment [5].

ESC offer an interesting model for the study of DNA replication due to their rapid cell cycle, short G1 phase, and the absence of a proper G1/S checkpoint [6]. These challenging conditions expose ESC to a constant state of replication stress (RS), a condition in which the progression of the DNA replication forks is hampered and may lead to fork collapse, with deleterious effects on genomic stability [7]. However, ESC are known to have a remarkably stable genome. This suggests the presence of ESC-specific mechanisms to counteract the effects of RS, most of which are currently unknown.

ESC in vitro harbor a small population of cells expressing genes specific for the 2-cells-stage of mouse development [5, 8]. These are known as 2C-like cells (2CLC) and possess the ability to differentiate in both embryonic and extraembryonic tissues. In our previous work, we observed that subjecting ESC to RS causes a massive transcriptional dysregulation [9]. Among the overexpressed genes we identified a group of 2-cells stage-specific genes and retroelements. Interestingly, the activity of the Serine/Threonin kinase ATR (Ataxia Telangiectasia and Rad3-related), the master regulator of RS response, proved to be necessary for the 2-cell stage genes activation [9]. This corresponds to a higher contribution to extraembryonic tissues when RS-affected ESC are injected into developing morulae [9]. The increase in extraembryonic contribution is in part dependent on the expression of the gene *Dux*, a master regulator of 2-cell-stage expression [9, 10].

In this work we investigated the transcriptional effects of RS by showing that among the genes controlled by RS are present many key TE development regulators including *Cdx2*, *Gata3*, *Eomes* and *Elf5* [11–13]. The expression of these genes is not limited to the time of RS administration and has long-term effects on ESC developmental capabilities and cell fate regulation.

## Results

### RS induces the expression of TE-related genes in ESC

**Fig. 1:**
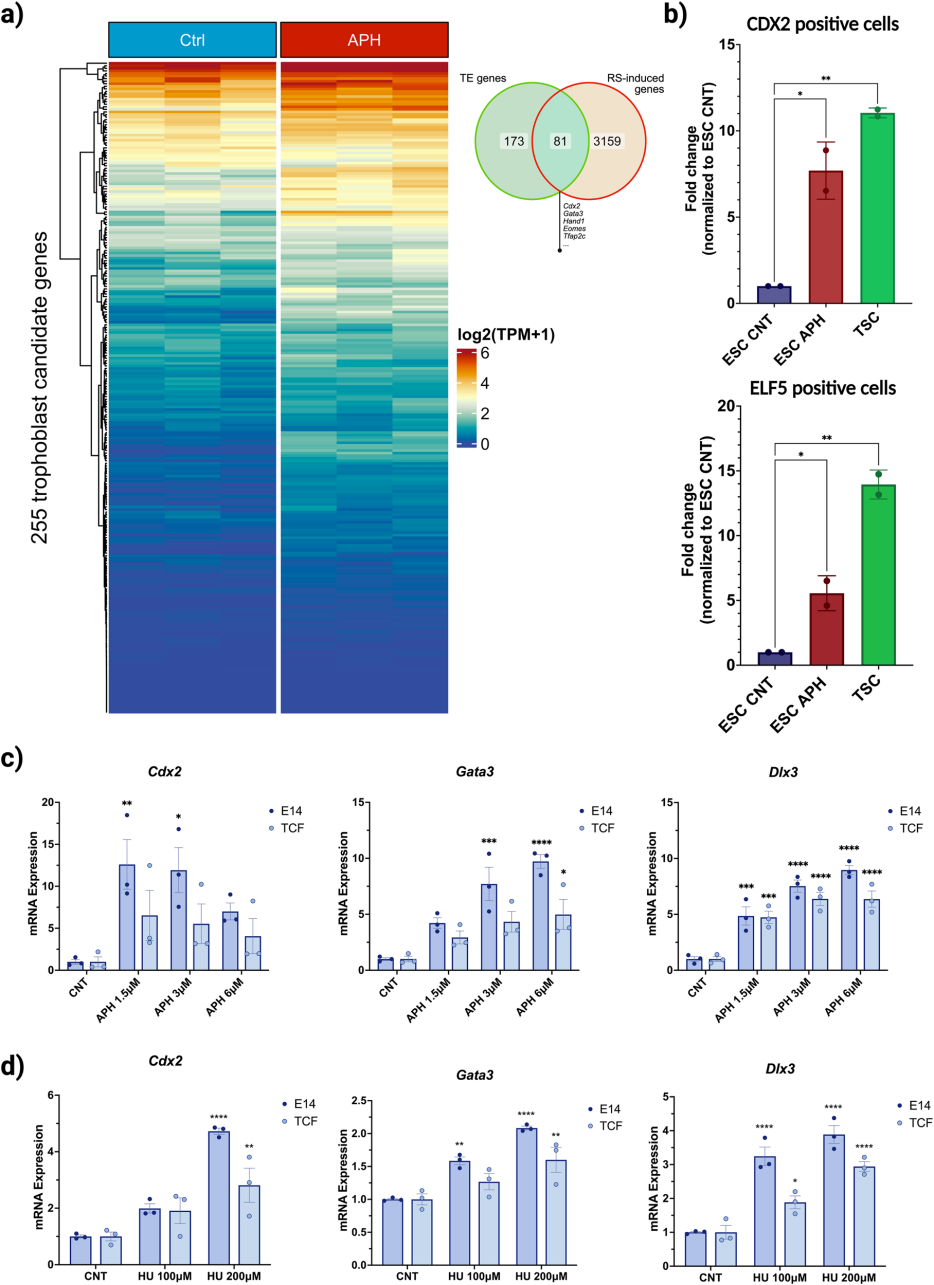
RS activates TE-specific genes. **a** Heatmap representing all TE-specific genes affected by RS in ESC. The Venn diagram shows the overlap between TE-specific genes and all the genes affected by RS. **b** Flow cytometry analysis of ESC expressing either CDX2 (top) or ELF5 (bottom) in control (CNT) conditions or after aphidicolin 3 µM treatment (APH). Trophoblast stem cells (TSC) are used as a control. Data represented as mean +/– SEM and analysed by One-way ANOVA (*n* = 2). **c** RT real-time-qPCR analysis for the expression of the indicated genes in E14 and TCF ESC lines either in control (CNT) conditions or treated with the indicated concentrations of aphidicolin (APH). **d** RT real-time-qPCR analysis for the expression of the indicated genes in E14 and TCF ESC lines either in control (CNT) conditions or treated with the indicated concentrations of hydroxyurea (HU). Data is expressed as fold change with respect to CNT for each cell line and normalized on *Tbp* expression. Data is represented as mean +/– SEM and analysed by One-way ANOVA (*n* = 3). For all the bar plots significance is indicated as follows: * = *p*-value < 0.05, ** = *p*-value < 0.01, *** = *p*-value < 0.001, **** = *p*-value < 0.0001.

In our previous work, we demonstrated that ESC undergo a dramatic transcriptional response in conditions of RS. Treatment of different ESC lines with aphidicolin (APH), an inhibitor of DNA polymerase α known to cause RS [14], resulted in the up-regulation of a large subset of genes, as we identified by bulk RNA-seq [9]. Our previous observation that RS favors ESC participation to the extraembryonic compartment led us to focus on the regulation of TE genes upon RS. In depth analysis of our RNA-seq dataset revealed several TE-specific genes among the ones affected by RS (Fig. 1a). To define a list of bona fide TE-related genes, we used published transcriptomic data comparing trophoblast stem cells (TSC) to ESC in vitro [15]. These genes were then crossed with a list of validated TE-expressed genes from the MGI database [16, 17], resulting in a set of 255 TE-specific genes (supplementary table 1). Among these genes, 81 (32%) were significantly upregulated by RS in our RNA-Seq data, including *Cdx2*, *Gata3*, *Eomes*, *Hand1* and *Tfap2c* (Fig. 1a). This contrasted with an overlap of only 14.9% in the case of a published set of primitive endoderm genes (supplementary table 2) [18]. These data were confirmed in four ESC lines (E14, TCF, R1 and BALB/c) by real-time RT-qPCR. Both lines, treated with increasing concentrations of APH, displayed a concentration-dependent increase in the expression of key TE genes (Fig. 1c and supplementary fig. 1c). The expression of TE regulators at the protein level was confirmed by flow cytometry, using antibodies against CDX2 and ELF5. Cells were treated with APH at 3 μM for 16 h or kept unperturbed as a negative control. Unperturbed TSC were used as a positive control. RS administration increased the number of both CDX2 and ELF5 expressing cells (Fig. 1b). The same result was observed when our four ESC lines were treated with another RS-inducing agent, namely hydroxyurea (HU). Treatment with increasing concentrations of HU led to activation of various TE-specific genes (Fig. 1d and supplementary fig. 1d).

### RS favors the differentiation of ESC toward a TE-like phenotype

**Fig. 2:**
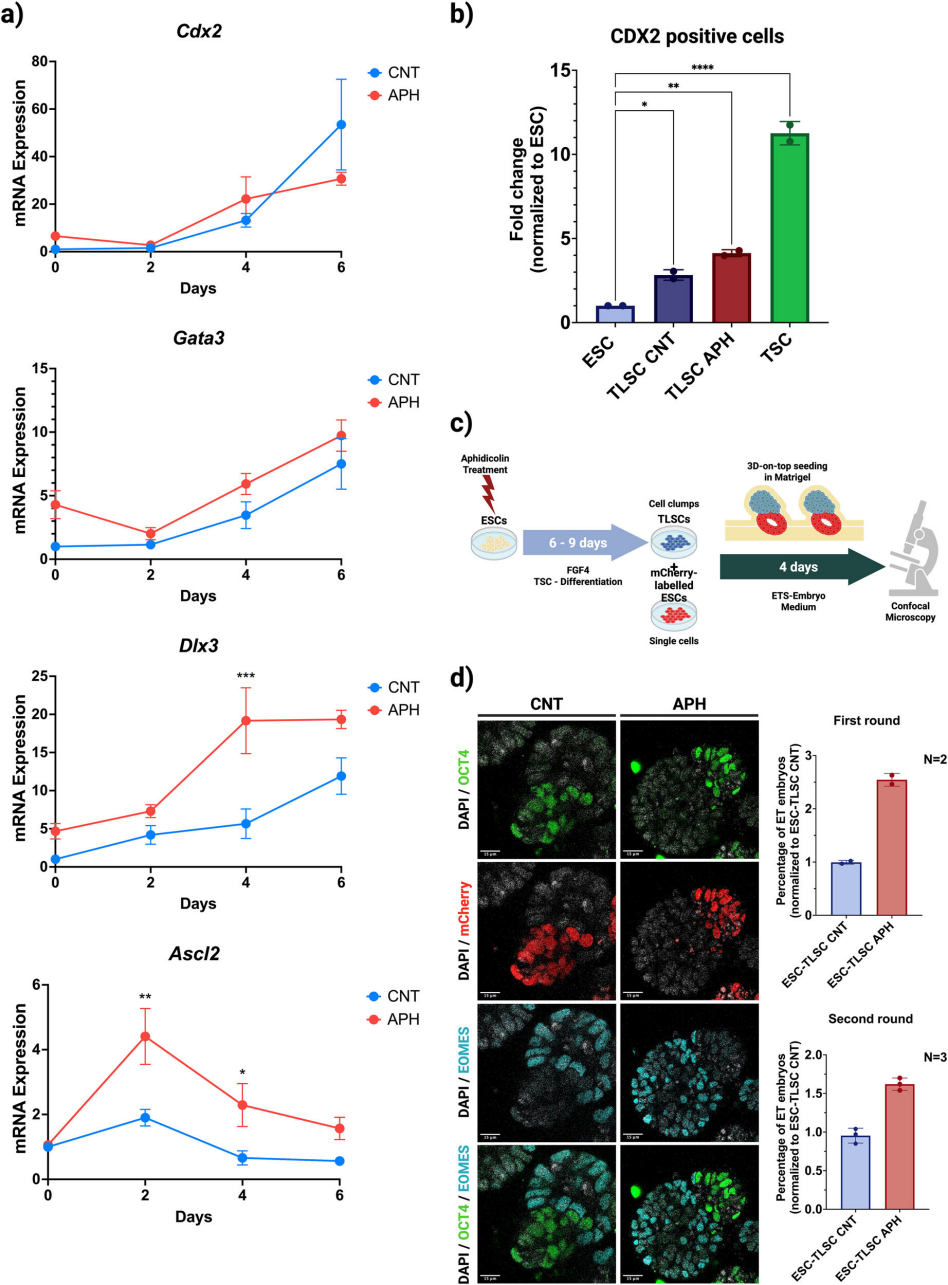
RS promotes TE differentiation capacity. **a** RT real-time-qPCR analysis for the expression of the indicated genes in cells kept either in control (CNT) conditions or subjected to aphidicolin 3 µM treatment (APH) before differentiation for the indicated number of days. Data is expressed as fold change with respect to day 0 CNT and normalized on *Tbp* expression. Data is represented as mean +/– SEM and analysed by Two-way ANOVA (*n* = 4). **b** Flow cytometry analysis of TLSC expressing CDX2 after FGF4-induced differentiation from ESC in control (CNT) conditions or after aphidicolin 3 µM treatment (APH). Unperturbed ESC and TSC are used respectively as negative and positive controls. Data represented as mean +/– SEM and analysed by One-way ANOVA (*n* = 2). **c** Scheme representing the experimental procedure followed to obtain embryoid structures. **d** Representative images (left) and quantification (right) of two rounds of embryoid structures generation starting from TLSC obtained from ESC kept in control conditions (CNT) or subjected to aphidicolin 3 µM treatment (APH) before differentiation, as shown in (**c**). For the bar plot, significance is indicated as follows: * = *p*-value < 0.05, ** = *p*-value < 0.01, *** = *p*-value < 0.001.

To evaluate the long-term effects of RS on ESC differentiation, we decided to monitor TE gene expression for several days after the source of stress was removed. To do so, we treated ESC with APH at 3 μM for 16 h. At the end of treatment (day 0), we washed away APH and kept the cells in a differentiation-permissive medium for 6 further days. ESC are typically maintained in an undifferentiated state through the use of leukemia inhibitory factor (LIF) and inhibitors targeting the MAP kinases and GSK pathways (2i medium) [19, 20]. To allow differentiation, ESC were cultured in a medium without these factors. As shown in Fig. 2a, the increase in early TE genes (*Cdx2* and *Gata3*) [11, 13, 21] expression due to RS was lost by day 2 after 2i and LIF withdrawal, and was then increased by the absence of the inhibitors in both conditions. On the contrary, the late TE genes *Ascl2* and *Dlx3* [22, 23] showed an increased expression in response to RS after 2 and 4 days post-removal of 2i and LIF (Fig. 2a). These data indicate that exposure to RS leads to long-term effects in ESC even after the stress has ceased. ESC have been shown to acquire a TE-like phenotype if cultured in a TSC medium containing FGF4 and heparin, acquiring both a TSC-like morphology and expressing key TE genes [24]. Therefore, we wondered if cells subjected to RS could be more receptive to FGF4-induced differentiation. To test this hypothesis, ESC were differentiated in TE medium for 6 days after APH treatment and analyzed by flow cytometry using an antibody against CDX2. Unperturbed ESC and TSC were used, respectively, as a negative and positive control. After differentiation into TE-like stem cells (TLSC), both conditions displayed an increase in the number of CDX2-expressing cells following TE differentiation in respect to unperturbed ESC (Fig. 2b). Noticeably, RS prior to differentiation induced a sensibly higher number of CDX2-expressing cells in comparison to unperturbed cells (Fig. 1b), confirming that RS favors TE differentiation even after several days since the removal of the stress.

To further confirm that RS improves ESC differentiation towards an extraembryonic phenotype, both cells, unperturbed or subjected to RS, were kept in TE medium to induce differentiation into TLSC. These cells were then used to generate embryoid structures by 3D co-culture with mCherry-labeled ESC (Fig. 2c). It has been in fact demonstrated that TSC co-cultured with aggregates of ESC are able to generate structures that recapitulate the characteristics of a blastocyst, with ESC constituting the ICM and the TSC composing the extraembryonic compartment [25, 26]. Although to a much lower extent, TLSC also proved to be able to form these embryoid structures, with an EOMES-marked “extraembryonic” compartment and an OCT4/mCherry-marked “embryonic” compartment (Fig. 2d and supplementary fig. 2b–d). Interestingly, TLSC generated from cells affected by RS showed a higher propensity to produce embryoids with a correct separation of the two compartments (Fig. 2d), suggesting again that RS may favor the acquisition of an extraembryonic phenotype.

### The expression of TE genes in response to RS is controlled by ATR activity

**Fig. 3:**
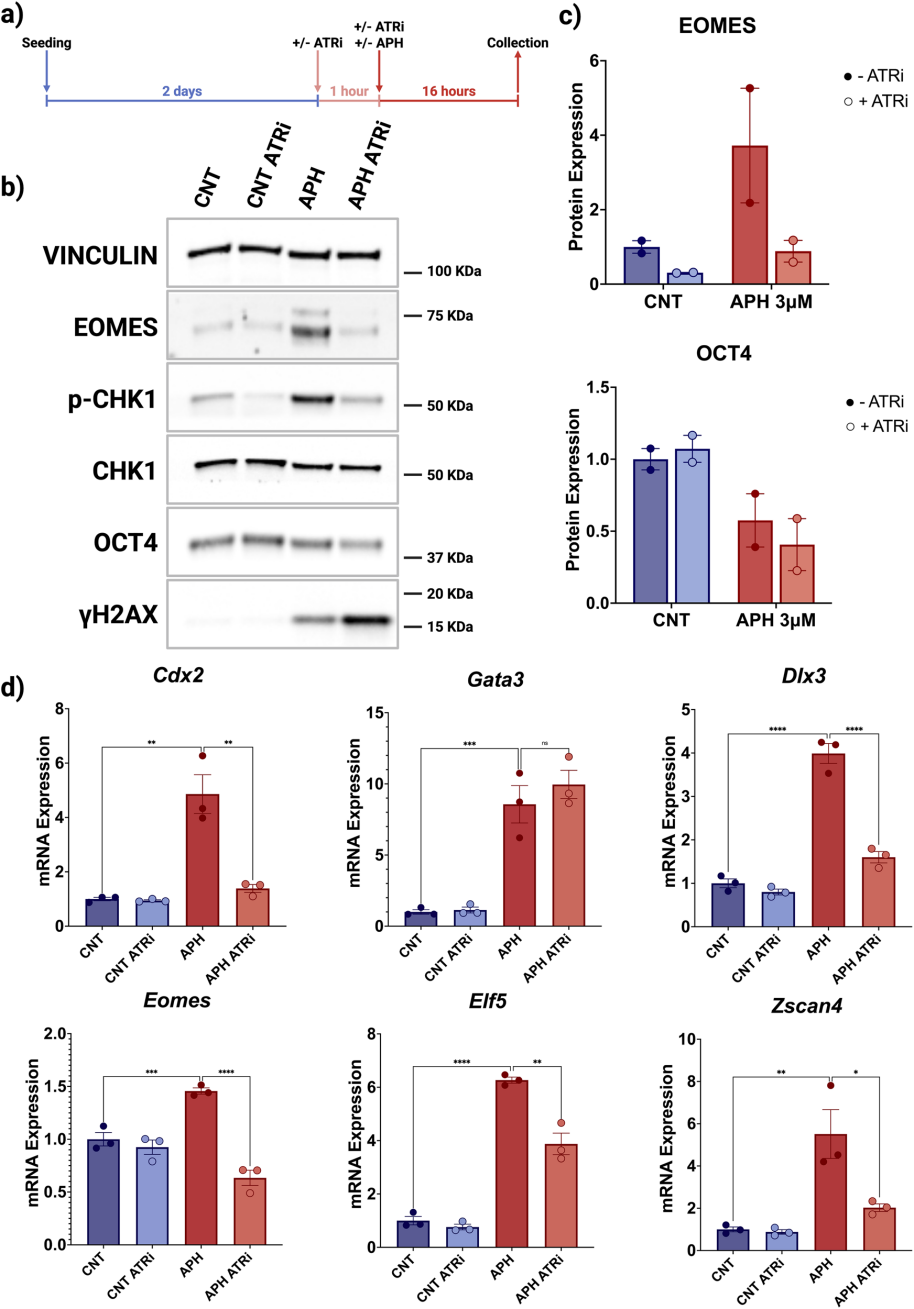
RS-induced expression of TE genes is ATR-dependent. **a** Schematic representation of the ATR inhibition experiment. **b** Representative image of a western blot analysis showing the expression level of the indicated proteins in cells subjected to aphidicolin 3 µM treatment (APH) or not (CNT) in combination with ATR inhibitor treatment (ATRi). The original images used to assemble this picture can be found as supplementary material 1. **c** Quantification of EOMES and OCT4 protein levels in the conditions represented in Fig. 3b. Each sample is normalized on a VINCULIN internal control and on CNT, and expressed as mean +/– SEM (*n* = 2). **d** RT real-time-qPCR analysis for the expression of the indicated genes for cells either in control (CNT) conditions or treated with aphidicolin 3 µM (APH) in combination or not with ATR inhibitor (ATRi). Data is expressed as fold change with respect to CNT -ATRi and normalized on *Tbp* expression. Data is represented as mean +/– SEM and analysed by One-way ANOVA (*n* = 3). For all the bar plots significance is indicated as follows: * = *p*-value < 0.05, ** = *p*-value < 0.01, *** = *p*-value < 0.001.

Given ATR’s role in activating 2 C genes in response to RS [9], we asked whether its activity is similarly required for TE gene expression in analogous conditions. Therefore, we treated ESC with APH at the concentration of 3 μM for 16 h, with or without 1 h pre-treatment and continued exposure to the ATR inhibitor VE-822 (Fig. 3a). Western blot analysis confirmed ATR inhibition by showing reduced RS-dependent CHK1 phosphorylation (Fig. 3b and supplementary material 1). This correlated with the suppression of RS-induced EOMES protein upregulation (Fig. 3b, c). Notably, the pluripotency marker OCT4, encoded by the gene *Pou5f1*, decreased at the protein level in response to RS (Fig. 3b, c), differently from what we observed at the mRNA level (supplementary Fig. 1b, c). However, ATR inhibition did not rescue OCT4 levels but reduced them further (Fig. 3b, c), indicating that TE gene expression in response to RS is not solely due to OCT4 downregulation. As expected, APH increased γH2AX, a marker of RS and DNA damage [27], which was further elevated by ATR inhibition in combination with RS (Fig. 3b). This was likely due to ATM-mediated H2AX phosphorylation, which is strongly activated by the formation of DNA double-strand breaks (DSB) following replication fork collapse in the absence of ATR activity [7]. Interestingly, this shows that TE gene expression in response to RS cannot be a result of replication fork collapse and DSB formation, but it is linked to the RS checkpoint activation.

Inhibition of ATR activity suppressed RS-induced gene expression also at the mRNA level, as measured by real-time RT-qPCR. Expression of TE key regulators *Cdx2*, *Elf5*, *Eomes* and *Dlx3*, which was induced by RS, was reduced by ATR inhibition, mirroring the behavior of the 2-cell-stage gene *Zscan4* (Fig. 3d). Interestingly, *Gata3* mRNA levels showed no response to ATR inhibition, suggesting that different TE genes may be subjected to distinct regulatory circuits (Fig. 3d).

### The activation of TE genes and 2-cell-stage genes in response to RS constitutes distinct phenomena

**Fig. 4:**
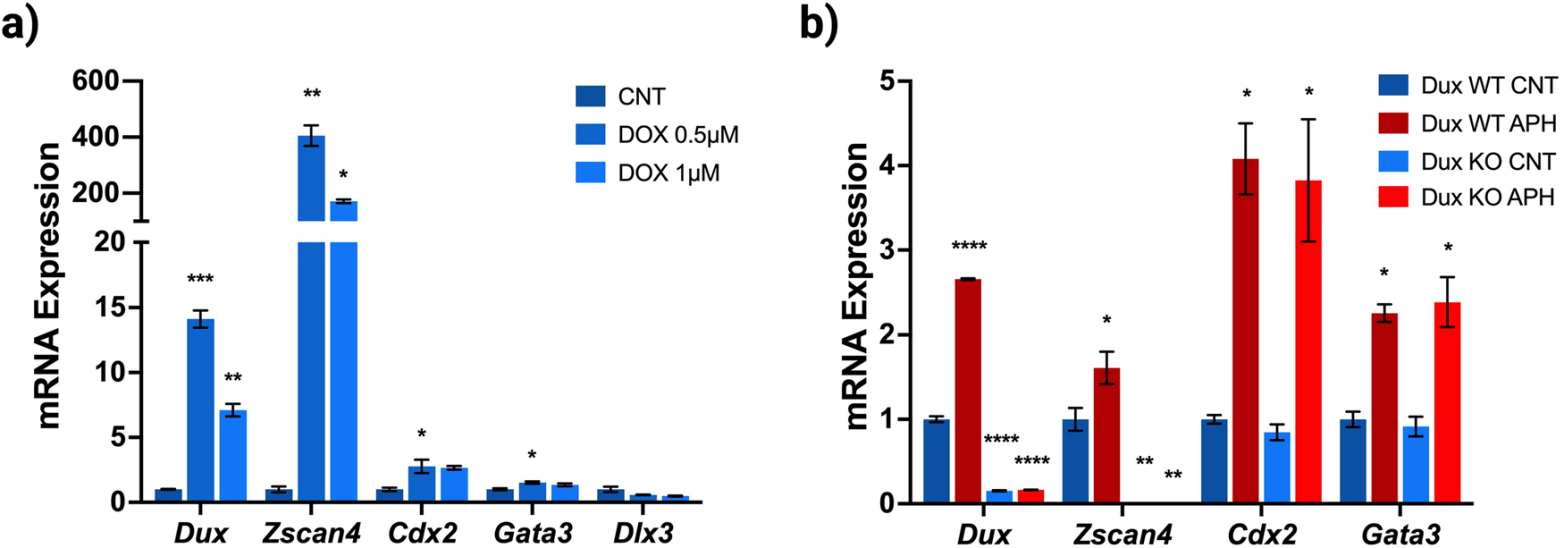
DUX is not required for RS-dependent expression of TE genes. **a** RT real-time qPCR analysis for the expression of the indicated genes for *Dux* inducible ESC treated with the indicated concentrations of doxycycline (DOX). Data is expressed as a fold change of CNT for each gene independently and normalized on *Tbp* expression. Data is represented as mean +/– SEM and analysed by One-way ANOVA (*n* = 3). **b** RT real-time qPCR analysis for the expression of the indicated genes for Dux WT and Dux KO lines, either in control (CNT) conditions or subjected to treatment with aphidicolin 3 µM (APH). Data is expressed as fold change of Dux WT CNT for each gene independently and normalized on *Tbp* expression. Data is represented as mean +/– SEM and analysed by One-way ANOVA (*n* = 3). For all the bar plots, significance is indicated as follows: * = *p*-value < 0.05, ** = *p*-value < 0.01, *** = *p*-value < 0.001, ****= *p*-value < 0.0001.

Since both TE and 2-cell-stage genes are up-regulated by RS in an ATR-dependent manner, we wondered if DUX, the main regulator of the 2-cell-stage program, could contribute to TE gene expression upon RS induction. This hypothesis is supported by the finding that a 3xHA-tagged DUX construct binds many TE-associated promoters when overexpressed in an ESC model, as demonstrated by Hendrickson et al. [10]. To test this hypothesis, we measured the expression of TE transcripts in an ESC line harboring a doxycycline-inducible *Dux* gene [9]. Doxycycline administration resulted in strong *Dux* expression at the mRNA level, mirrored by a similarly strong up-regulation of its main target *Zscan4* (Fig. 4a). However, this did not correspond to an increase in the expression of TE genes, such as *Cdx2*, *Gata3*, *Dlx3*, *Eomes* and *Elf5* (Fig. 4a). Although *Cdx2* and *Gata3* expression mildly increased upon doxycycline administration, this up-regulation did not match the concomitant massive activation of the 2-cell stage gene *Zscan4*, suggesting that their increase was not a direct effect of the 2-cell stage program activation. Since *Dux* overexpression proved insufficient to activate TE gene expression, we wondered if it could still be necessary for their RS-dependent activation. To test this hypothesis, we employed a Dux knockout (Dux KO) ESC line generated via CRISPR/nickase and its isogenic wild-type control (Dux WT) [28]. Treating the Dux WT line with APH 3μM resulted in up-regulation of both 2-cell-stage genes, such as *Dux* itself and *Zscan4*, and TE genes, such as *Cdx2* and *Gata3* (Fig. 4b). On the contrary, in Dux KO cells, no expression of both *Dux* and *Zscan4* was observed (Fig. 4b). However, APH treatment of Dux KO ESC activated TE gene expression to levels comparable to Dux WT cells (Fig. 4b), indicating that *Dux* is not required for RS-dependent expression of TE genes.

### Mass spectrometry analysis unveils a significant loss of proteins from ESC chromatin upon RS

**Fig. 5:**
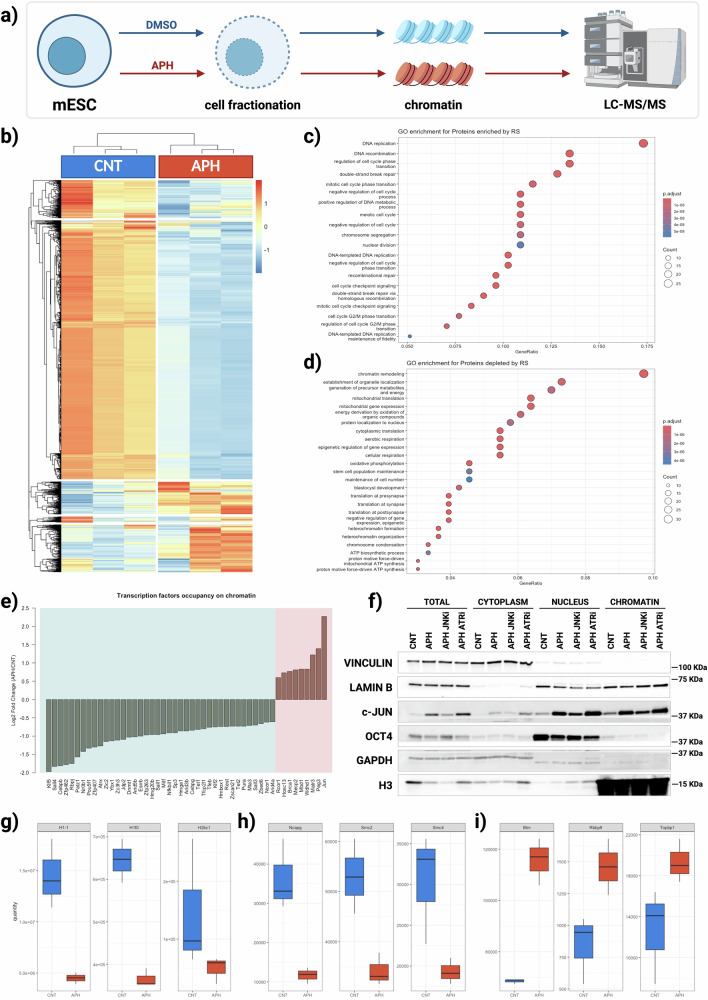
RS induces ESC chromatin remodelling and c-Jun accumulation. **a** Scheme representing the experimental procedure followed to obtain chromatin proteomic data. **b** Heatmap depicting the relative level of proteins found on the chromatin of ESC either kept in control conditions (CNT) or treated with aphidicolin 3 µM (APH) for three different biological replicates. **c** Gene ontology enrichment analysis of genes corresponding to proteins enriched on the chromatin of ESC subjected to RS. **d** Gene ontology enrichment analysis of genes corresponding to proteins depleted from the chromatin of ESC subjected to RS. For both (**d, e**) dots, colour represent significance as measured by adjusted *p*-value and dots represent the number of genes associated to each GO term. **e** Bar plot showing the enrichment level of several transcription factors on the chromatin of ESC subjected to RS expressed as fold change over CNT. **f** Western blot showing the expression level of the indicated proteins in different subcellular fractions for the indicated conditions. ESC were kept in control conditions (CNT), treated with aphidicolin 3 µM (APH), treated with aphidicolin and JNK inhibitor (APH+JNKi) or aphidicolin and ATR inhibitor (APH+ATRi). The original images used to assemble this picture can be found as supplementary material 2. **g** Box plot showing the presence in the chromatin of different histones in CNT or APH conditions. **h** box plot showing the presence of condensin components in the chromatin in CNT or APH conditions. **i** Box plot showing the presence of different DNA damage response proteins in CNT or APH conditions.

To investigate the mechanisms underlying TE genes activation upon RS, we analyzed the chromatin composition in ESC under normal conditions and after 16 h of APH 3 μM treatment by mass spectrometry (Fig. 5a). The analysis found that a large group of proteins was depleted from ESC chromatin in response to RS, while a markedly lower number was enriched (Fig. 5b). Among these proteins, 362 showed a significant downregulation while 165 displayed a significant increase. Depleted proteins belong to gene ontology (GO) terms such as chromatin organization, heterochromatin organization and chromosome condensation (Fig. 5d), suggesting that RS reduces the compaction of ESC chromatin. At the same time, also terms related to epigenetic regulation and blastocyst development were affected by RS, confirming the massive dysregulation of ESC identity (Fig. 5d). In particular, among depleted proteins we observed several nucleosome subunits, such as histones H1.1, H1f0, H2B type 1-A and others, but also subunits of the condensin complex (SMC2, SMC4 and NCAPG) (Fig. 5g, h and supplementary table 3) [29]. Conversely, enriched proteins were associated with GO terms linked to DNA repair and DNA damage response (Fig. 5c). RPA subunits and the BRCA1 protein accumulated on the chromatin in response to the APH treatment, as expected by their role in sensing RS [30, 31]. Accordingly, the ATR-binding protein TOPBP1 displayed a significant increase in response to RS, together with proteins involved in homologous recombination, such as BLM, RBBP8, RAD52 and others (Fig. 5i and supplementary table 3) [32–34].

To identify potential TE regulators among RS-affected proteins, we crossed the list of dysregulated proteins with transcription factors identified by the FANTOM5 project [35]. While 40 transcription factors showed a significant reduction in chromatin, only 9 displayed an increase (Fig. 5e). Pluripotency-associated transcription factors, like OCT4, KLF5, SALL4 and ESRRB, were lost from the chromatin, confirming that RS interferes with ESC cell identity [36–38]. Among the enriched transcription factors we observed some repressors, such as RCOR1, MECP2 and MBD1, the transcription factor PEG3, involved in both p53 and TNF pathways, and the stress-responsive protein c-JUN, part of the AP-1 complex [39, 40] (Fig. 5e). Importantly, western blot analysis confirmed the accumulation of c-JUN on chromatin in response to RS, which was mostly driven by an increase in its expression in response to RS (Fig. 5f and supplementary material 2). This increase was reduced by the inhibition of the JNK family of kinases (using the specific inhibitor SP600125), involved in c-JUN pathway activation. ATR inhibition instead had no effect on c-JUN levels (Fig. 5f).

### The JNK/c-JUN pathway favors the expression of TE genes in response to RS

**Fig. 6:**
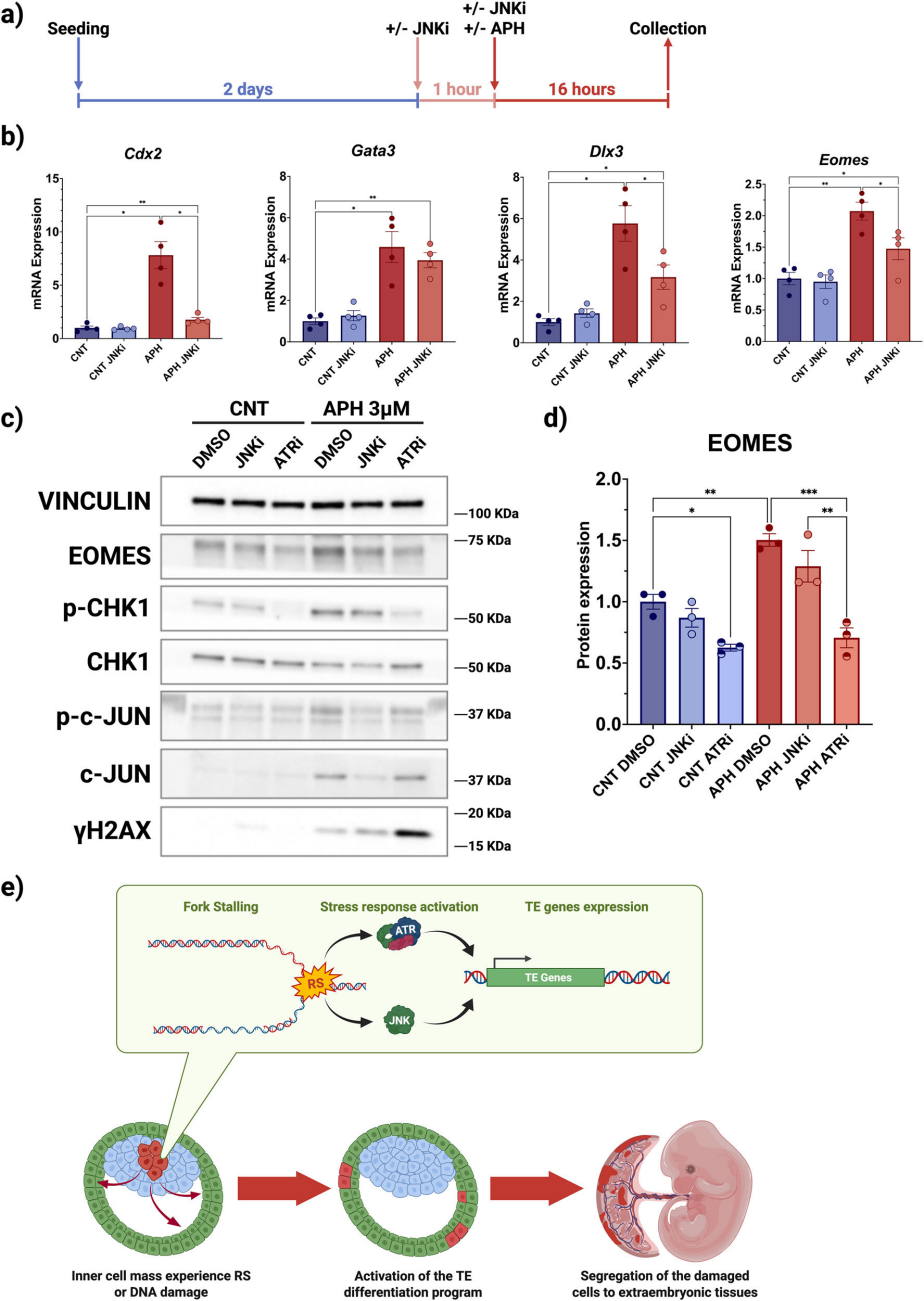
The JNK/c-Jun axis promotes TE genes expression upon RS. **a** Schematic representation of the JNK inhibition experiment. **b** RT real-time-qPCR analysis for the expression of the indicated genes for cells either in control (CNT) conditions or treated with aphidicolin 3 µM (APH) in combination or not with JNK inhibitor (JNKi). Data is expressed as fold change in respect to CNT -JNKi and normalized on *Tbp* expression. Data is represented as mean +/– SEM and analysed by One-way ANOVA (*n* = 4). **c** Representative western blot showing the protein expression levels of the indicated proteins and the phosphorylation levels of CHK1 (p-CHK1), c-JUN (p-c-JUN) and histone H2AX (γH2AX) in the indicated conditions. The original images used to assemble this picture can be found as supplementary material 3. **d** Quantification of EOMES protein levels in the conditions represented in Fig. 6b. Each sample is normalized on a VINCULIN internal control and on CNT, and expressed as mean +/– SEM (*n* = 3). Data was analysed by One-way ANOVA. **e** Scheme representing our current working model. RS affecting cells of the inner cell mass during development can induce the differentiation of cells toward extraembryonic lineages.

Since c-JUN displayed a consistent RS-induced accumulation on chromatin and is a major player in cell fate regulation, we wondered if this could be related to TE gene expression in RS conditions. To test this hypothesis, ESC were pre-treated for 1 h with a JNK inhibitor, followed by 16 h with or without APH 3 μM, keeping JNK inhibition throughout (Fig. 6a). JNK inhibition markedly reduced APH-induced expression of key TE genes *Cdx2*, *Dlx3*, and *Eomes*, but not *Gata3* (Fig. 6b), suggesting that these genes are regulated by both ATR and JNK pathways during RS. Interestingly, EOMES protein was less sensitive to JNK inhibition, while ATR inhibition displayed a strong suppression (Fig. 6c, d and supplementary material 3). As expected, JNK inhibition decreased c-JUN phosphorylation on threonine 91 (T91), a marker of AP-1 activation [41], without affecting CHK1 phosphorylation on serine 317 (S317), a marker of ATR activation (Fig. 6c). Notably, inhibition of JNK led to a reduction in the level of the protein itself (Fig. 6c), indicating that efficient activation of the JNK/c-JUN pathway is important for the increase in c-JUN protein expression itself.

## Discussion

DNA replication is a fundamental core cellular process. Adverse events affecting it may result in extensive mutations and even challenge the survival of the cell. This acquires particular significance during the early stages of embryonic development, when mutations affecting a relatively small number of cells can result in their transmission to entire tissues or organs with the progression of development. Eliminating damaged cells through apoptosis may not always be compatible with the survival of the embryo, which is composed of very few cells at the beginning of development. The prevalence of massive mutations in the placenta [42, 43], even when the embryo proper shows an intact genome, suggests that segregating cells subjected to high levels of stress to the extraembryonic compartment could be a potential mechanism to preserve the genomic integrity of the embryo without sacrificing a large number of cells (Fig. 6e).

Our previous work highlighted the surprising ability of ESC to acquire a less differentiated phenotype in response to RS thanks to the expression of the transcription factor DUX [9]. In this work, we showed how even TE-specific genes are activated in response to RS, indicating that RS does not direct ESC toward a specific fate, but leads them to acquire different, developmentally close phenotypes. Similarly to 2C-specific genes, also TE genes display a dependency on the RS master regulator ATR [7], although their expression does not require the transcription factor DUX. This indicates that while RS induces in ESC the expression of both 2 C and TE genes, the two phenomena are controlled distinctly. Surprisingly, TE gene induction by RS is independent of TEAD4, the main effector of the Hippo pathway during TE development [44] (supplementary fig. 2d, e). This suggests that TE gene activation in response to RS does not follow the normal mechanisms of TE development.

Interestingly, other groups have proved that hyperosmotic stress leads to the activation of extraembryonic endoderm genes in ESC cultivated in the absence of 2i [45]. In this case, the stress-induced expression is controlled by the MEK1/2, c-Jun/JNK and PI3K pathways [45]. Interestingly, also in our case, JNK proved to be important for TE gene expression in response to RS. It is well known that c-JUN and other AP-1 complex components are activated in response to DNA damage and other stresses with various outcomes [39, 40]. Naïve ESC were reported to have very low levels of *c-Jun* expression that increase with the development progression [46]. In addition, it has been proven that *c-Jun* acts as a barrier in cell reprogramming [47]. Interestingly, we show that *c-Jun* levels can be increased in ESC under replicative stress conditions. According to our data, the activation of the JNK kinases induces the expression of TE genes, indicating that the increased presence of c-JUN across more differentiated cell types may not be simply a characteristic of the later stages of development but may have an active role in the progression of differentiation. However, it is important to notice that the AP1 transcription factor, downstream to JNK activity, assumes a large variety of compositions involving multiple members of the c-JUN and FOS families. At this stage, it’s not clear which members of the family are required for the activation of TE expression.

Withdrawal of 2i and LIF promoted the expression of early TE genes to a greater extent than RS alone. Nevertheless, the expression of late-stage TE genes, such as *Dlx3* and *Ascl2*, displayed higher levels in cells treated with APH in comparison to the control, even several days after the removal of the source of stress. In addition, when exposed to a differentiation medium containing FGF4, RS-affected ESC showed a higher propensity to take part in the formation of embryoid structures and overall higher expression of TE markers. This is in line with our previous finding that RS-affected ESC are more likely to terminally differentiate towards trophoblast giant cells in vitro, and to participate in the formation of extraembryonic tissues when injected in morula stage embryos [9]. Taken together, these results indicate that the effects of RS are not limited to the acute phase of stress but result in long-lasting changes in cell identity that may have important consequences in determining cell fate. The importance of a phenomenon allowing cells to acquire a TE-related phenotype in response to RS is not limited to embryonic development. It is well known that many solid tumors experience significant levels of RS due to their unregulated proliferation [48, 49]. A connection between RS and the acquisition of TE features could suggest a way by which tumors could develop hallmarks such as vasculogenesis, invasiveness or immune evasion capabilities, which are very well-known characteristics of the placenta [50]. Nevertheless, further studies are required to explore the similarities between the acquisition of TE-features during development and tumorigenesis, and how RS affects cell identity and phenotypic plasticity.

In conclusion, this work proves a new way in which perturbations in the normal course of DNA replication induce changes in cell identity, resulting in the redirection of the cell towards an extraembryonic fate. This process is dependent on the activation of stress-responsive pathways such as the ATR and JNK pathways (Fig. 6e). This shows that the role of stress-responsive mechanisms is not limited to cell protection or induction of cell death, but may lead to drastic rearrangements of cell identity, affecting the developmental abilities of the cell.

## Materials and methods

### Cell culture

E14 and TCF ESC were grown in feeder-free conditions in 2i medium composed as follows: KnockOut DMEM (ThermoFisher, 10829-018), 15% ESC qualified Fetal Bovine Serum (ThermoFisher, 16141-079), 2mM L-glutamine, 0.1 mM non-essential amino acids, 0.1 mM β -mercaptoethanol, 50units/mL penicillin, 50 mg/mL streptomycin, 1000 units of leukemia inhibitory factor (LIF ESGRO, Sigma ESG1107), 1 μM PD-0325901 (Axon Medchem, 1408) and 3 μM CHIR-99021 (Axon Medchem, 1386). Cells were cultured in low oxygen conditions. For each experiment, ESC were seeded 48 h before collection at 15,000 cells/cm^2^. Cells were treated with aphidicolin (Sigma, A0781) at various concentrations, the ATR inhibitor VE-822 (Axon Medchem, 2452) at the concentration of 1 μM, the JNK inhibitor SP 600125 at the concentration of 25 μM, or their combination. Doxycycline for the induction of *Dux* expression was administered at 0.5 μM or 1 μM. Embryo-derived mouse TSC, a kind gift from Janet Rossant's lab (The Hospital for Sick Children, Canada), were cultured in TSC medium: 30% RPMI 1640 (Lonza, BE12-167F) supplemented with 20% ESC qualified FBS (ThermoFisher, 10829-018), 1 mM pyruvate, 2 mM L-glutamine, 100 mM β-Mercaptoethanol, 70% of conditioned medium from mitomycin-C-inactivated mouse embryonic fibroblasts (MEFs), 25 ng/mL FGF4 (R&D Systems, 235-F4-025) and 1 μg/mL heparin (Sigma-Aldrich, H3149).

### ESC in vitro differentiation toward TE lineages

ESC were differentiated toward TSC as previously reported [12, 24, 51]. Briefly, ESC subjected or not to RS were cultured in TSC medium starting from 24 h after the end of treatment and kept in these conditions from 6 to 9 days. Medium was changed daily, and cells were split once every 2–3 days. In alternative cells were incubated after treatment for up to 6 days in medium without LIF and the two inhibitors PD-0325901 and CHIR-99021 to allow ESC differentiation.

### *Dux* OE and *Dux* KO cell lines

*Dux* overexpressing ESC (*Dux* OE) were generated according to Atashpaz et al. [9]. The *Dux* knock-out (*Dux* KO) line and its isogenic control (*Dux* WT) were a kind gift of Alberto De Iaco and Didier Trono (School of Life Sciences, École Polytechnique Fédérale de Lausanne (EPFL), Lausanne, Switzerland) [28].

### Transfection and esiRNA-mediated knockdown

Cells were transfected at seeding using Lipofectamine RNAiMax (Invitrogen). The knockdown was performed by endoribonuclease-prepared small interfering RNAs (esiRNAs), a pool of different dsRNAs covering a 500p long stretch on the target mRNA. The ribonucleoparticles targeting *Tead4* (Sigma-Aldrich, EMU083251) were assembled in OptiMEM (Thermofisher) and added to the cells at a final concentration of 60 nM. ESCs were then incubated at 37 °C under 3% O2 tension and 5% CO_2_ tension for 48 h before sample collection.

### RNA extraction, cDNA synthesis and real-time qPCR

RNA was extracted using the RNeasy Mini Kit (QIAGEN, 74104) after removal of DNA contaminants by DNAse I digestion (QIAGEN, 79254). cDNA was prepared from 1 to 2 μg of total RNA using the iScript kit (BioRad, 1708890) using a random primer-based approach.

The real-time qPCR assay was performed with SsoFastTM EvaGreen® Supermix (Bio Rad, 1725201), 0.5 μM primer mix and 5 ng of sample cDNA in technical triplicate. The *Tbp* gene was used as a normalizer to analyze the data following the ΔΔCt method. Data was analyzed using either the one-way ANOVA with Tukey’s post-test correction or the unpaired two-tailed T-test functions of the GraphPad Prism software (GraphPad). The list of used primers can be found in supplementary table 4.

### RNA-seq data analysis

RS-induced genes were obtained from Atashpaz et al. [9] (NCBI BioProject PRJNA415135). The RNA-seq count matrix of TSC and ES samples was downloaded from GSE189683149. Genes for which the sum of their counts across samples was < 10 were eliminated. DESeq2 (v1.38.3) was used for differential expression analysis with “design = ~ replicate + tissue” and “alpha=0.05”. 7030 upregulated genes were identified (log2 fold change > 1 and FDR < 0.05) and then cross-referenced with 671 TE genes queried from the mouse genome informatics (MGI) database, resulting in 255 TE-specific genes, 81 of which are upregulated in response to RS. The expression profiles of TE-specific genes in APH vs CNT ESC were examined by plotting their log2(TPM + 1) values in a heatmap using ComplexHeatmap (v2.14.0) in R.

### Protein extraction and western blot

Cell pellets were lysed in RIPA buffer (NaCl 150 mM, NP-40 1%, Sodium deoxycholate 0.5%, Sodium dodecyl sulphate 0.1%, and Tris pH7.4 25 mM in water) supplemented with a protease/phosphatase inhibitor cocktail (Cell Signaling, 5872) for 30 min at 4 °C. Samples were subsequently sonicated with a Bioruptor Sonication System (UCD200) at 4 °C to complete lysis. 20 μg of each sample was loaded on 4–15% precast gels (BioRad). Proteins were then transferred to nitrocellulose membranes by a semi-dry approach (Trans-Blot Turbo Transfer, BioRad). Filters were blocked with either 5% milk in TBST buffer (Tris pH 7.4, 20 mM, NaCl 150 mM, 0.1% Tween) or 5% BSA in TBST (for detection of phosphorylated proteins) for 1 h at room temperature. After blocking, filters were incubated with primary antibodies overnight at 4 °C on rotation and washed before incubation with HRP-conjugated secondary antibodies for 1 h at room temperature. Filter images were acquired using the Thermofisher iBright system. The following antibodies were used: anti-p-CHK1 (S317) (Cell signaling, 12302), anti-CHK1 (Santa Cruz, sc8408), anti-GAPDH (Sigma-Aldrich, G8795), anti-EOMES (Abcam, ab233345), anti-OCT4 (Santa Cruz, sc-5279), anti-c-JUN (Cell signaling, 9165), anti-p-c-JUN (T91) (Abcam, ab79756), anti-γH2AX (Millipore, 05-636), anti-VINCULIN (Sigma-Aldrich, V9131), anti-LAMIN B1 (Abcam, ab16048) and anti-H3 (Cell signaling, 4620).

### Cell fractionation and chromatin isolation

Cell fractionation for both western blot analysis and mass spectrometry was performed using the Subcellular Protein Fractionation Kit (ThermoFisher, 78840). Briefly, cell pellets washed with ice-cold PBS were resuspended in CEB buffer for 10 min at 4 °C, in order to permeabilize cellular membranes, and then centrifuged at 500 × *g* for 5 min to collect the cytoplasmic fraction (supernatant). Cellular membranes were subsequently solubilized in MEB buffer for 10 min at 4 °C after intense vortexing and then collected by centrifugation at 3000 × *g* for 5 min (supernatant). Pellets were then resuspended in NEB buffer and vortexed for 15 s and then incubated for 30 min at 4 °C to free the nuclear content. Supernatants containing the nuclear fraction were then collected by centrifugation for 5000 × *g* at 5 min. NEB buffer supplemented with 5 mM CaCl2 and 300 units of Micrococcal Nuclease was added to the remaining pellets at room temperature, followed by vortexing for 15 s. The mixes were then incubated 15 min at room temperature to digest DNA, and the freed chromatin fractions were collected by centrifugation for 5 min at 16000 × *g*. The cytoplasmic, nuclear and chromatin fractions were analyzed by western blot, while chromatin fractions were also subjected to mass spectrometric analysis.

### Sample preparation for mass spectrometry

Frozen chromatin fractions were manually homogenized in a buffer containing 8 M urea and 100 mM Tris-HCl (pH 8.0). To aid in complete solubilization, samples underwent five cycles of tip sonication (on/off). The lysates were then centrifuged at 14,000 × *g* for 30 min at 4 °C, and protein concentrations in the resulting supernatants were determined using the Microplate BCA™ Protein Assay Kit (ThermoFisher). For each sample, 50 μg of total protein was subjected to standard in-solution digestion. Briefly, proteins were reduced with 10 mM tris(2-carboxyethyl) phosphine (TCEP; ThermoFisher) and alkylated with 40 mM 2-chloroacetamide (Sigma-Aldrich) in the presence of 8 M urea and 100 mM Tris-HCl (pH 8.0). The reaction was carried out simultaneously for 30 min at room temperature in the dark with gentle shaking. A two-step proteolytic digestion protocol was employed. Initially, endoproteinase Lys-C (ThermoFisher) was added at a 1:50 enzyme-to-protein ratio and incubated for 1 h at room temperature. Subsequently, the digestion was enhanced by adding trypsin (Roche) at the same ratio, followed by overnight incubation at 37 °C. The resulting peptide mixtures were desalted and concentrated using μ-C18 ZipTip pipette tips (Millipore), according to the manufacturer’s guidelines. Cleaned peptides were then reconstituted in 5% formic acid (FA) and stored at –20 °C until further analysis by mass spectrometry.

### MS acquisition and data processing

MS analysis was carried out using an Easy-nLC 1200 ultra-high-performance liquid chromatography (UHPLC) system (ThermoFisher) coupled to an Orbitrap Exploris 480 mass spectrometer (ThermoFisher). 2 μg of each peptide sample was loaded onto the system and separated using a linear gradient elution, from 95% solvent A (2% acetonitrile, 0.1% formic acid) to 55% solvent B (80% acetonitrile, 0.1% formic acid). Proteomic data were acquired using both Data-Dependent Acquisition (DDA) and Data-Independent Acquisition (DIA) modes. DIA raw data files were analyzed using Spectronaut software (version 15, Biognosys). For protein identification and quantification, a DDA library-assisted search was performed using the Pulsar search engine. Peptide spectra were matched against the UniProtKB 2020 complete mouse proteome database. For identification searching, methionine oxidation (M) and N-terminal acetylation (Protein N-term) were set as variable (dynamic) modifications, while carbamidomethylation of cysteine residues (C) was defined as a fixed (static) modification. Peptide assignment required a minimum length of seven amino acids, allowing up to two missed cleavages, in line with Lys-C and trypsin specificity. Data were filtered at a 1% false discovery rate (FDR) at the protein level to ensure high-confidence identifications. Quantitative protein analysis was performed using the integrated statistical tools available in Spectronaut. As a first step, likely contaminants and low-confidence identifications were removed. Signal intensities were normalized across runs and log₁₀-transformed. To address missing values, imputation was performed using the underlying MS signal from detected peaks. For statistical evaluation, an unpaired t-test was conducted to compare protein expression between experimental groups. Proteins were considered significantly differentially expressed if they met two criteria: a q-value (FDR-adjusted *p*-value) ≤ 0.05 and an absolute log2 fold change ≥ 0.58, corresponding to a 1.5-fold change in either direction.

### Flow cytometry

ESCs were fixed and permeabilized using the Cytoperm/Cytofix kit (BD Biosciences, 554714), and subsequently stained for 1 h at room temperature with conjugated primary antibodies. The following antibodies were used: anti-ELF5-Alexa Fluor 647 (G-Biosciences, ITA8395) and anti-CDX2-Alexa Fluor 488 (Santa Cruz, sc-393572). Cells were then washed and acquired on a FACSCalibur instrument (BD Biosciences, RRID: SCR_000401) or Attune NxT (Thermo Fisher Scientific).

### ET embryo generation

H2B-mCherry ESC colonies were dissociated into single cells, while TLSC were kept in small clumps by incubation in trypsin-EDTA. ESC pellets were washed with PBS twice to remove 2i. Cells were subsequently counted, and an equal number of ESC and TLSC were mixed and repelleted in ET-embryo medium (ETM) (Cell guidance systems, M13-100) [25, 26]. Then, the mixed cells were cultured on growth factor-reduced Matrigel (BD Biosciences, 356230) in μ-Slide 8 well plates (Ibidi, 80826) according to the 3D on-top approach for four days [52]. After 48 h of culture, the medium was changed every 12–24 h, depending on the speed of acidification of the medium and kept in culture for a total of 4 days. ET embryos were fixed with ice-cold 4% formaldehyde in PBS for 15 min at RT, then rinsed twice in PBS. Permeabilization was performed with 0.3% Triton-X-100 and 0.1% Glycine in PBS for 10 min at room temperature. Following, ET embryos were washed with PBS and incubated with primary antibodies overnight in blocking buffer (PBS plus 10% FBS and 1% Tween-20) at 4 °C. Cells were then washed twice in PBS and incubated overnight in secondary antibodies at 4 °C. DAPI in PBS (5 mg/ml) was added before imaging. Images were acquired with a Leica TCS SP2 AOBS inverted confocal microscope with 63X magnification. Acquired images were analyzed by ImageJ software (NIH, RRID: SCR_003070). For each condition, the number of structures showing the correct division between an mCherry-positive OCT4-marked compartment a mCherry-negative EOMES-expressing compartment were considered as ET embryos and divided by the total number of structures identified for that condition. The final results were expressed as a percentage.

### Statistical analysis

All experiments were performed in mouse embryonic stem cell lines (E14, TCF, R1, and BALB/c) as biological replicates, with each replicate derived from an independent cell passage and treatment. RT-qPCR assays were conducted in technical triplicate within each biological replicate. For RT-qPCR experiments, the exact sample size is indicated in each figure legend and corresponds to independent biological replicates (*n* = 3 or *n* = 4 as specified per experiment). Flow cytometry experiments were performed in *n* = 2 independent biological replicates. Western blot quantifications were performed in *n* = 2 or *n* = 3 independent biological replicates, as indicated per figure. Embryoid structure formation experiments were conducted over two independent rounds of structure generation. Mass spectrometry chromatin proteomic experiments were performed using *n* = 3 independent biological replicates. Given that some experiments were conducted with *n* = 2 replicates, which is below the threshold for meaningful descriptive statistics, individual data points are displayed for those datasets.

Statistical analyses were performed using GraphPad Prism software (GraphPad Software, La Jolla, CA). For RT-qPCR and flow cytometry data involving more than two groups, a one-way analysis of variance (ANOVA) was applied, with Tukey’s post-hoc test used to correct for multiple comparisons. Time-course differentiation experiments were analyzed by two-way ANOVA. All ANOVA tests were followed by Tukey’s multiple comparisons correction unless otherwise stated. For pairwise comparisons, an unpaired two-tailed Student’s *t* test was applied. All tests were two-sided. A *p*-value threshold of 0.05 was used to define statistical significance. Significance is indicated throughout the figures as follows: *p < 0.05, **p < 0.01, ***p < 0.001, ****p < 0.0001. For mass spectrometry data, statistical evaluation was performed using an unpaired t-test within Spectronaut software; proteins were considered significantly differentially expressed when meeting both a q-value (FDR-adjusted *p*-value) ≤ 0.05 and an absolute log₂ fold change ≥ 0.58 (corresponding to a 1.5-fold change). All center values reported in bar plots and quantifications represent the arithmetic mean. Error bars throughout all figures represent the standard error of the mean (s.e.m.).

## Supplementary information

Supplementary Figures

TE-specific genes list

PrE-specific genes list

Significantly variated chromatin proteins

List of oligos used in this study

Original immunoblots

## Data Availability

MS proteomic data are available through the PRIDE repository within the ProteomeXchange Consortium [53] using the dataset identifier PXD071534.
