# Supplementary material for: Replication stress primes a trophectoderm fate in embryonic stem cells
Source: Cell Death Discov. 2026 Jun 2;12:326. doi: 10.1038/s41420-026-03169-w (PMC13443534; doi:10.1038/s41420-026-03169-w)
Supplement: Supplementary file 1 [file 41420_2026_3169_MOESM1_ESM.pdf]

Supplementary Information

Replication stress primes a trophectoderm fate in embryonic stem cells

Andrea Gnocchi[1,2], Christelle El Kai[1], Cristina Castellan[1,2], Lucia Santorelli[1], Negar Arghavanifard[1,2], Angela Bachi[1] and Vincenzo Costanzo[1,2,*]

1 IFOM ETS - The AIRC Institute of Molecular Oncology, Milan, Italy

2 Hematology-Oncology department, University of Milan, Milan, Italy.

* Corresponding author

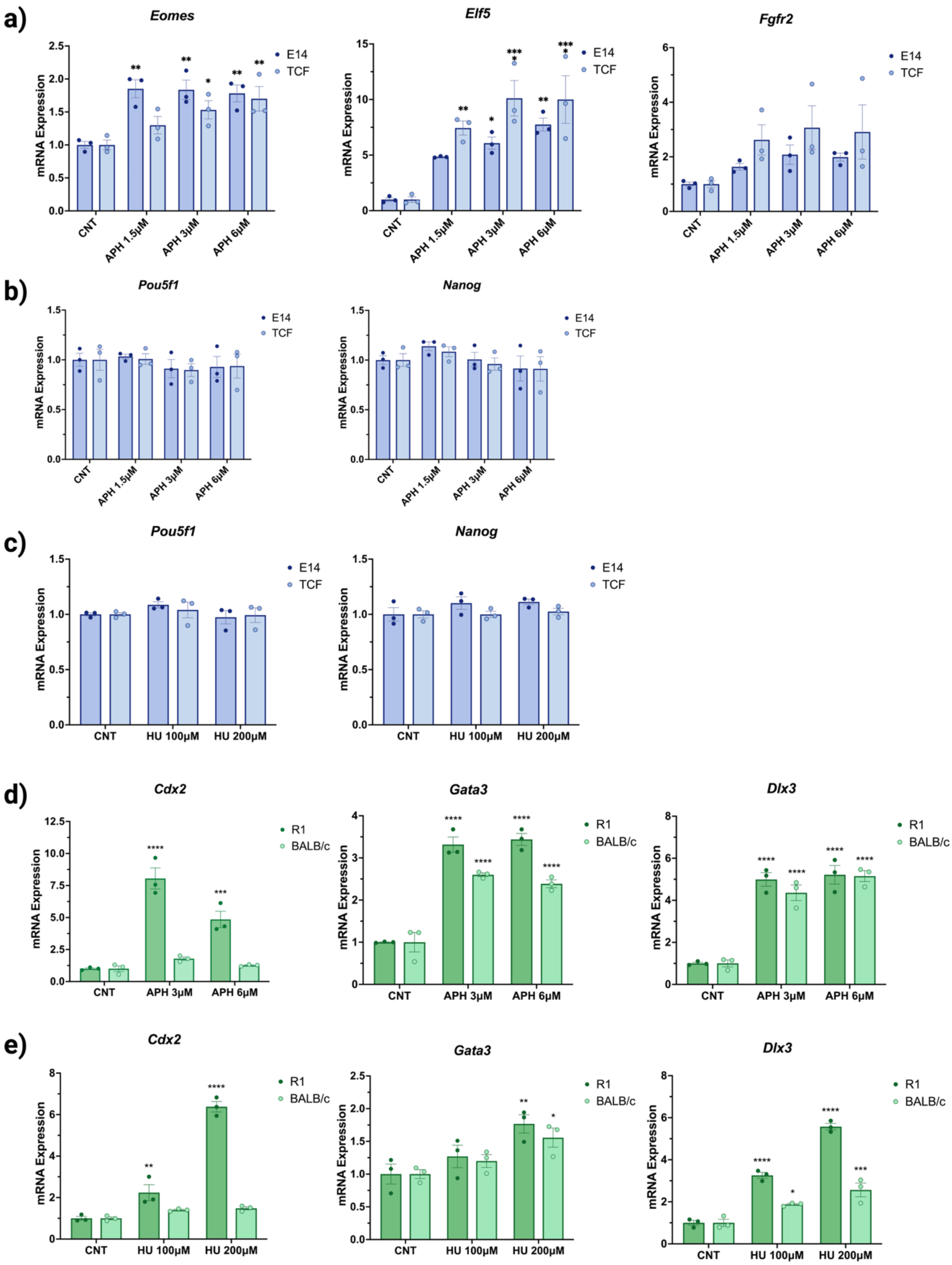
a)
Eomes
Elf5
Fgfr2
E14
TCF
mRNA Expression
CNT
APH 1.5μM
APH 3μM
APH 6μM
b)
Pou5f1
Nanog
E14
TCF
mRNA Expression
CNT
APH 1.5μM
APH 3μM
APH 6μM
c)
Pou5f1
Nanog
E14
TCF
mRNA Expression
CNT
HU 100μM
HU 200μM
d)
Cdx2
Gata3
Dlx3
R1
BALB/c
mRNA Expression
CNT
APH 3μM
APH 6μM
e)
Cdx2
Gata3
Dlx3
R1
BALB/c
mRNA Expression
CNT
HU 100μM
HU 200μM

Supplementary figure 1: a,b) RT real time-qPCR analysis for the expression of the indicated genes in E14 and TCF ESC lines either in control (CNT) conditions or treated with the indicated concentrations of aphidicolin (APH). c) RT real time-qPCR analysis for the expression of the indicated genes in E14 and TCF ESC lines either in control (CNT) conditions or treated with the indicated concentrations of hydroxyurea (HU). d,e) RT real time-qPCR analysis for the expression of the indicated genes in R1 and BALB/c ESC lines either in control (CNT) conditions or treated with the indicated concentrations of aphidicolin (APH) or hydroxyurea (HU). Data is expressed as fold change in respect to CNT for each cell line and normalized on *Tbp* expression. Data is represented as mean +/- SEM and analysed by One-way ANOVA (n=3).

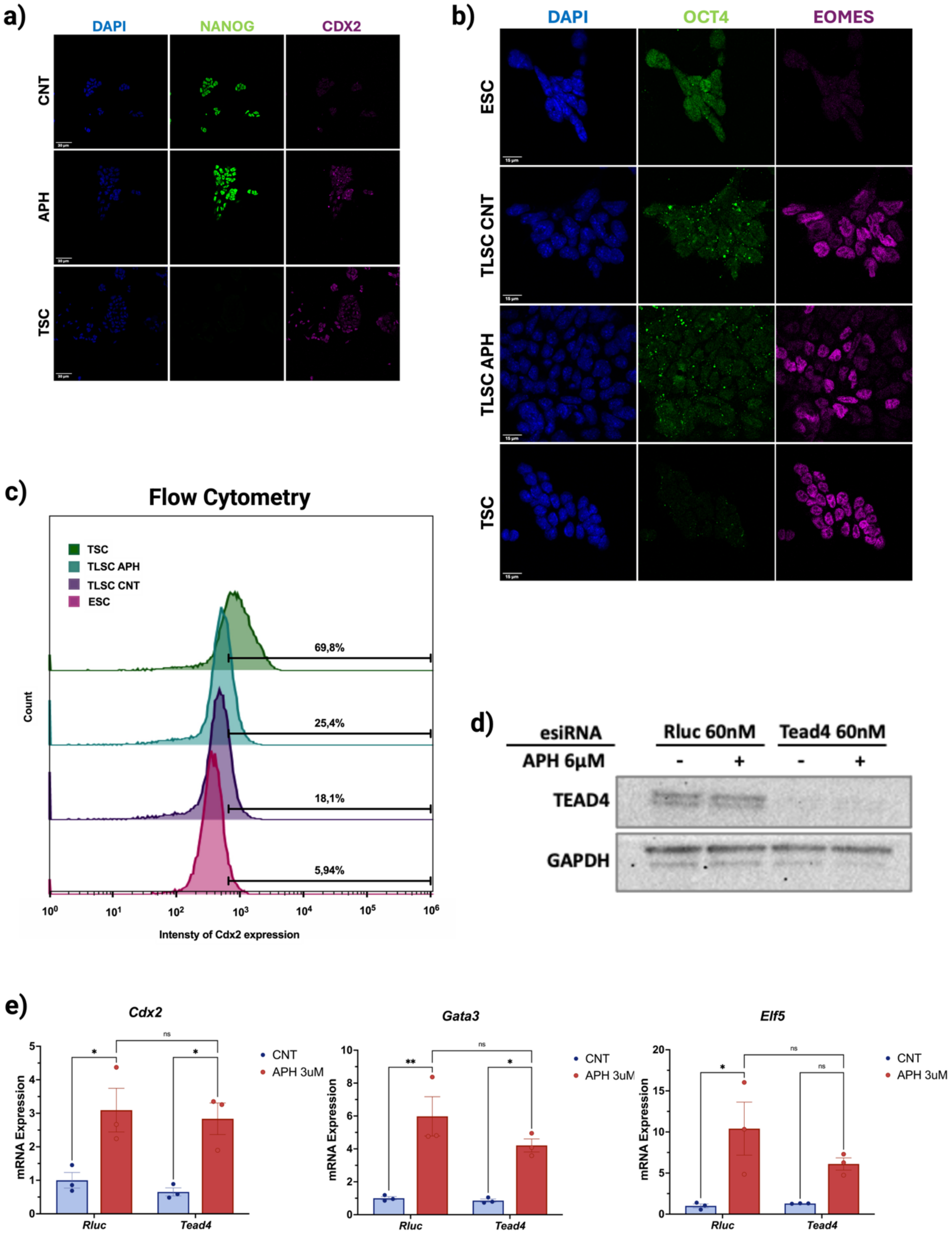

a)
DAPI
NANOG
CDX2
CNT
APH
TSC
b)
DAPI
OCT4
EOMES
ESC
TLSC CNT
TLSC APH
TSC
c)
Flow Cytometry
TSC
TLSC APH
TLSC CNT
ESC
69,8%
25,4%
18,1%
5,94%
Count
Intensty of Cdx2 expression
d)
esiRNA
Rluc 60nM
Tead4 60nM
APH 6μM
TEAD4
GAPDH
e)
Cdx2
Gata3
Elf5
mRNA Expression
CNT
APH 3uM
Rluc
Tead4
ns

Supplementary figure 2: a) Immunofluorescence analysis of ESC kept in control (CNT) conditions or subjected to aphidicolin (APH) treatment and stained for NANOG or CDX2 expression. Trophoblast stem cells (TSC) are used as a positive control. b) Immunofluorescence analysis of ESC kept in control (CNT) conditions or subjected to aphidicolin (APH) treatment before differentiation into TLSC and stained for OCT4 or EOMES expression. TSC and ESC are used as a negative and positive control. c) Representative density plot displaying the CDX2 expression relative to the flow cytometry analysis shown in figure 2b. d) Representative western blot showing the levels of TEAD4 protein in response to the esiRNA mediated knock-down of TEAD4 both in CNT conditions or in combination with APH treatment. GAPDH was used as a loading control. e) RT real time-qPCR analysis for the expression of the indicated genes in E14 ESC either in control (CNT) or aphidicolin (APH) conditions in combination with esiRNA mediated knock-down of TEAD4. Data is expressed as fold change in respect to CNT and normalized on *Tbp* expression. Data is represented as mean +/- SEM and analysed by One-way ANOVA (n=3).

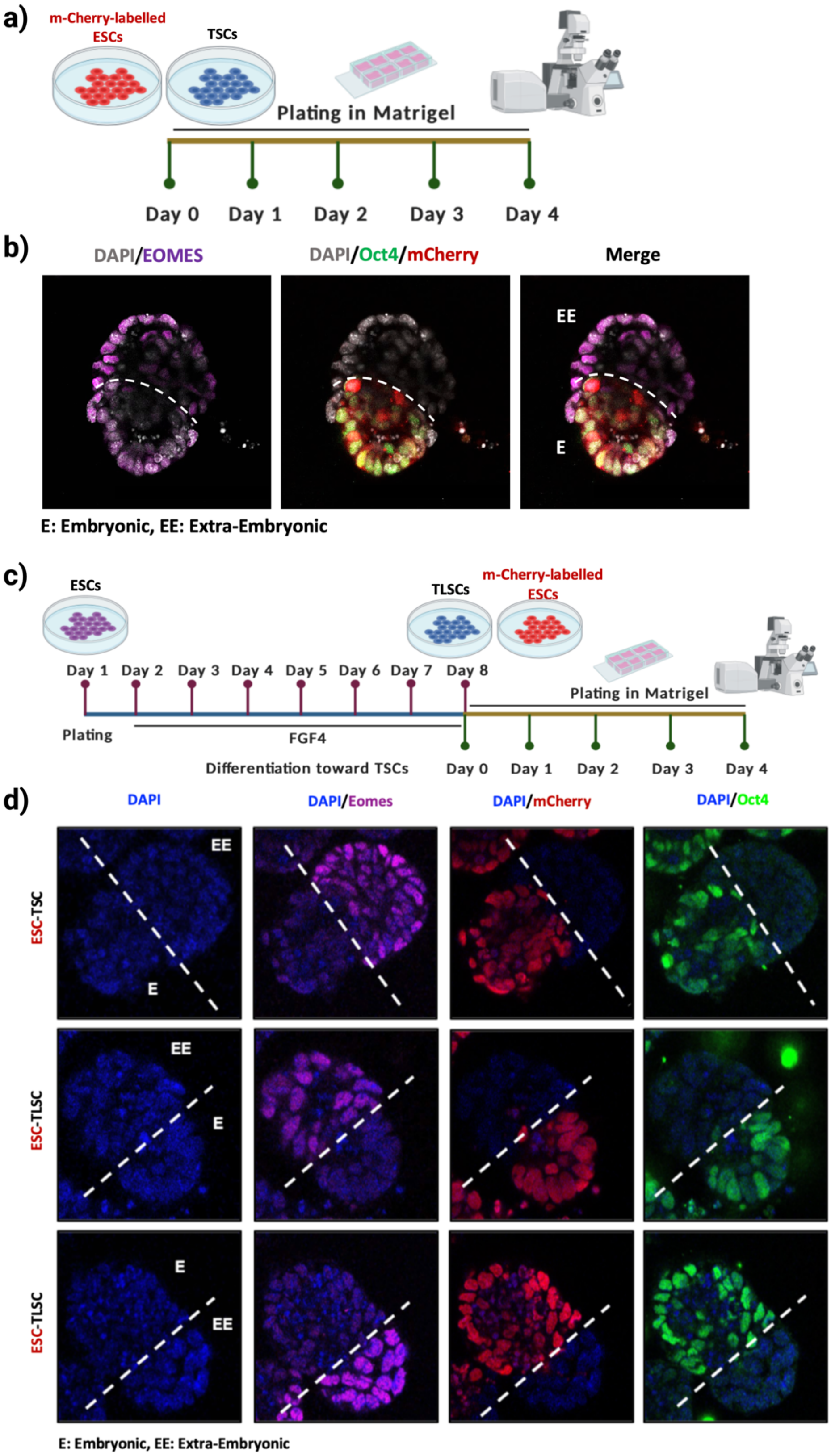
a)
m-Cherry-labelled ESCs
TSCs
Plating in Matrigel
Day 0
Day 1
Day 2
Day 3
Day 4
b)
DAPI/EOMES
DAPI/Oct4/mCherry
Merge
EE
E
E: Embryonic, EE: Extra-Embryonic
c)
ESCs
TLSCs
m-Cherry-labelled ESCs
Day 1
Day 2
Day 3
Day 4
Day 5
Day 6
Day 7
Day 8
Plating in Matrigel
Plating
FGF4
Differentiation toward TSCs
Day 0
Day 1
Day 2
Day 3
Day 4
d)
DAPI
DAPI/Eomes
DAPI/mCherry
DAPI/Oct4
ESC-TSC
ESC-TLSC
ESC-TLSC
EE
E
EE
E
E
EE
E: Embryonic, EE: Extra-Embryonic

Supplementary figure 3: a) Shematics showing the protocol used to produce embryoids by TSC and mCherry-labelled ESC. b) Representative immunofluorescence image of an embryoid generated as in supplementary figure 2a and stained with the indicated antibodies. EE: extra-embryonic compartment, E: embryonic compartment. c) Schematics showing the protocol used to produce embryoids starting from ESC differentiated with the FGF4 based protocol into TLSC. d) Immunofluorescence images of embryoids generated as in supplementary figure 2c and stained with the indicated antibodies. EE: extra-embryonic compartment, E: embryonic compartment.
