# Supplementary material for: Replication stress primes a trophectoderm fate in embryonic stem cells
Source: Cell Death Discov. 2026 Jun 2;12:326. doi: 10.1038/s41420-026-03169-w (PMC13443534; doi:10.1038/s41420-026-03169-w)
Supplement: Supplementary file 2 [file 41420_2026_3169_MOESM6_ESM.pdf]

Supplemental material

Replication stress primes a trophectoderm fate in embryonic stem cells

Andrea Gnocchi[1,2], Christelle El Kai[1], Cristina Castellan[1,2], Lucia Santorelli[1], Negar Arghavanifard[1,2], Angela Bachi[1] and Vincenzo Costanzo[1,2,*]

1 IFOM ETS - The AIRC Institute of Molecular Oncology, Milan, Italy

2 Hematology-Oncology department, University of Milan, Milan, Italy.

* Corresponding author

Original western blot images

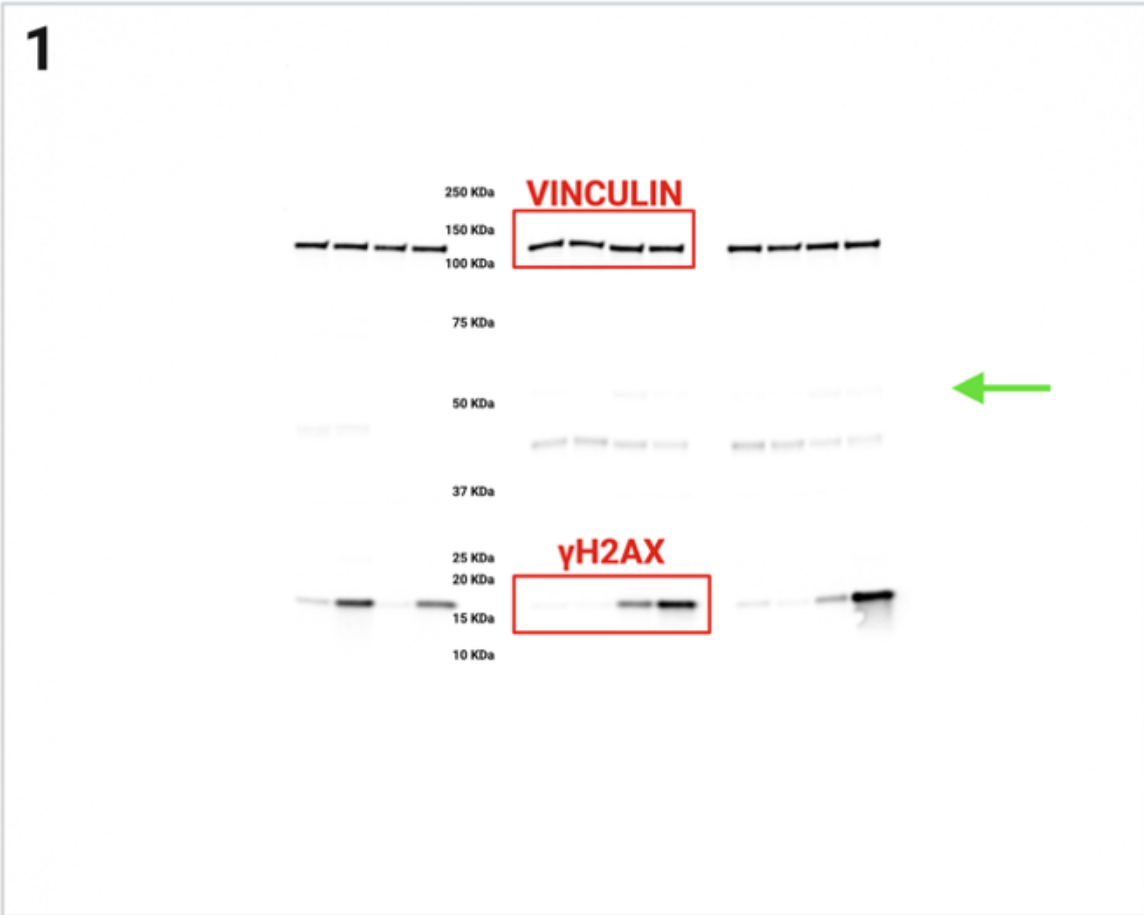
1
VINCULIN
γH2AX

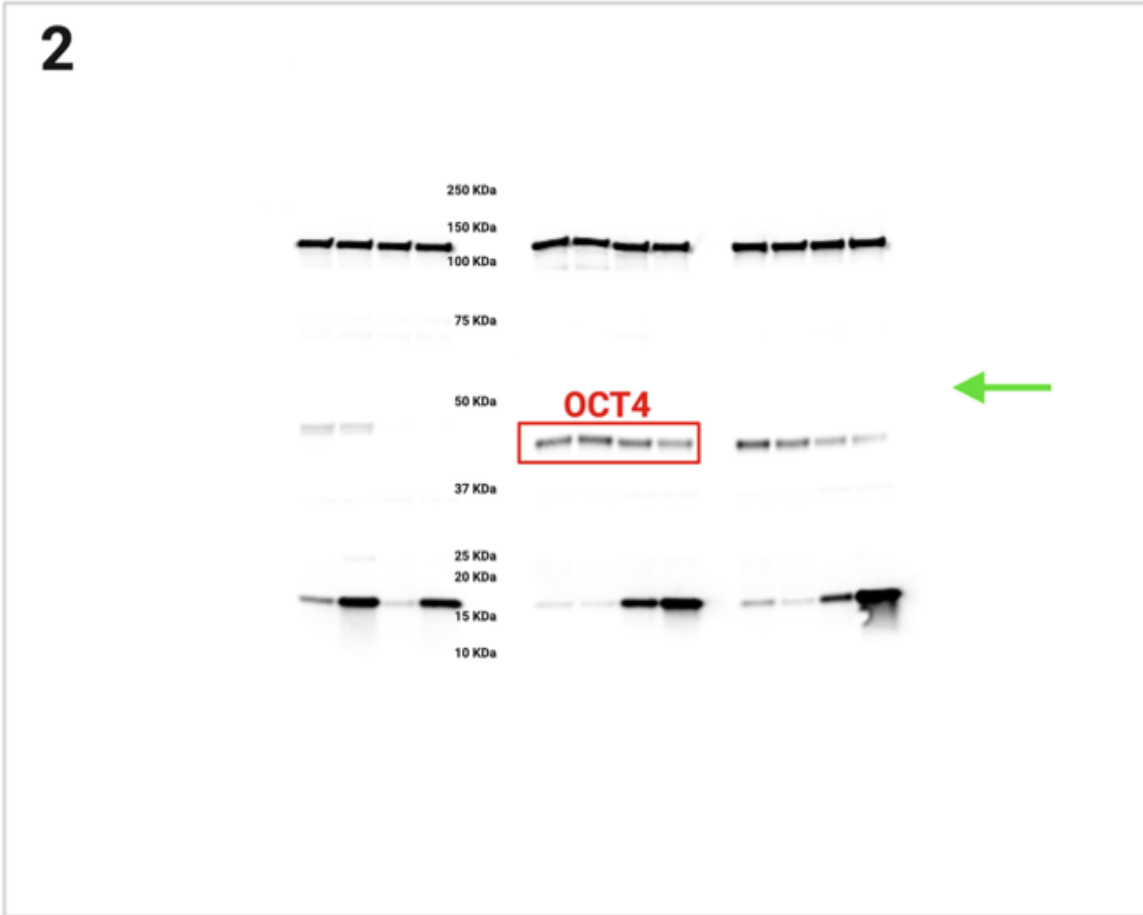
2
OCT4

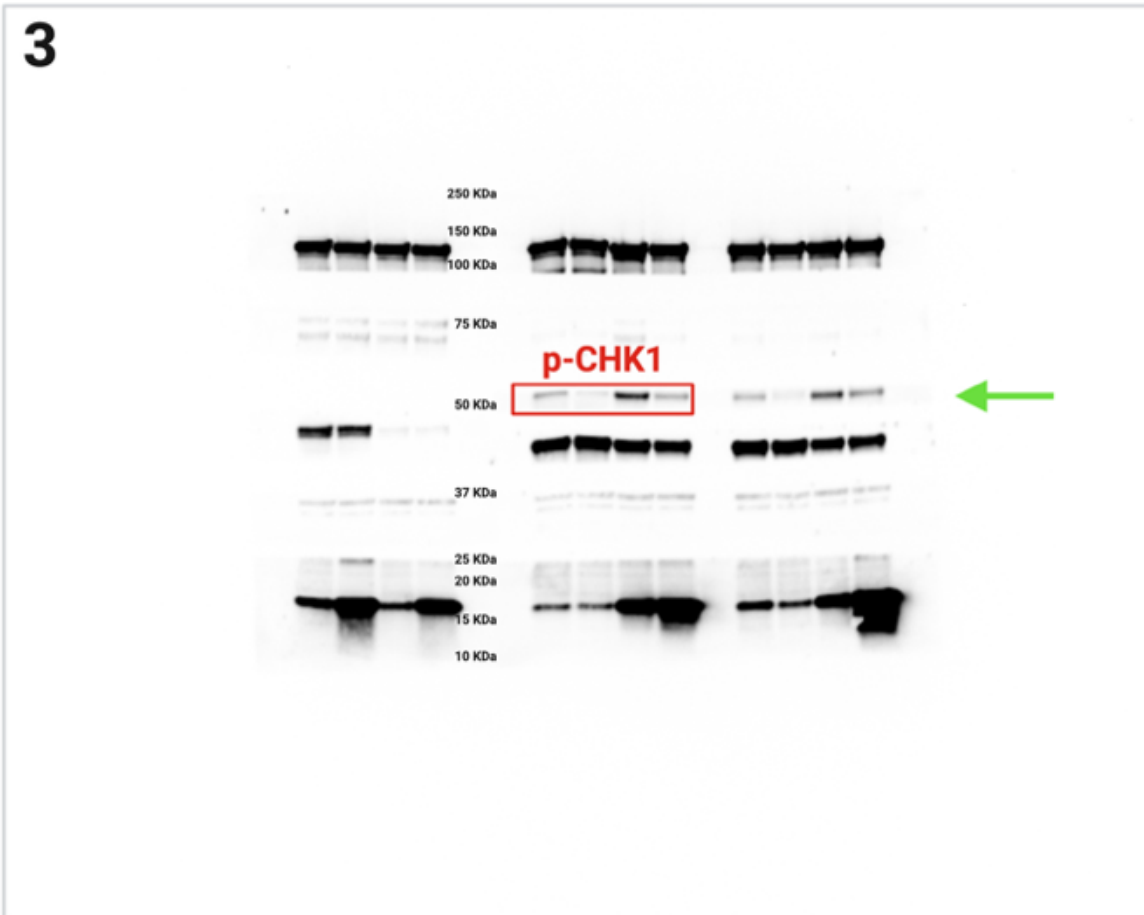
3
p-CHK1

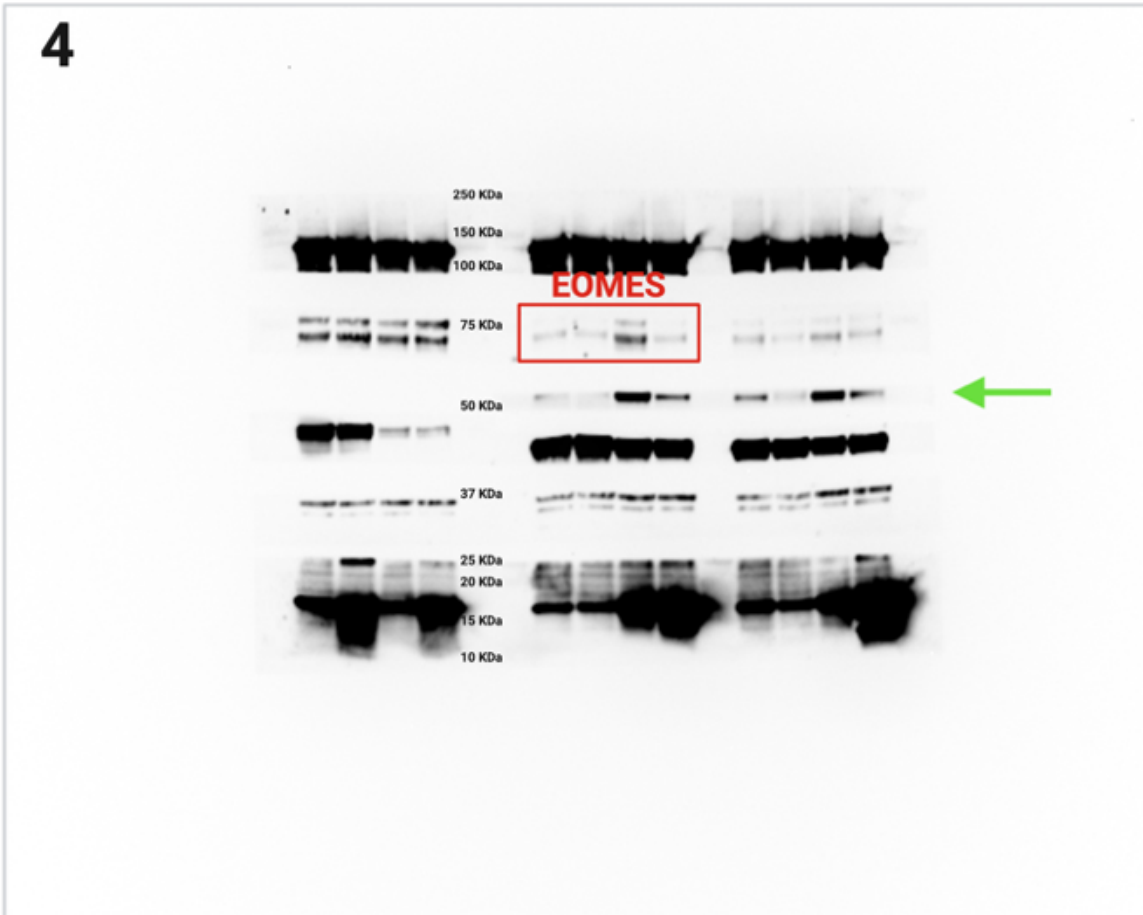
4
EOMES

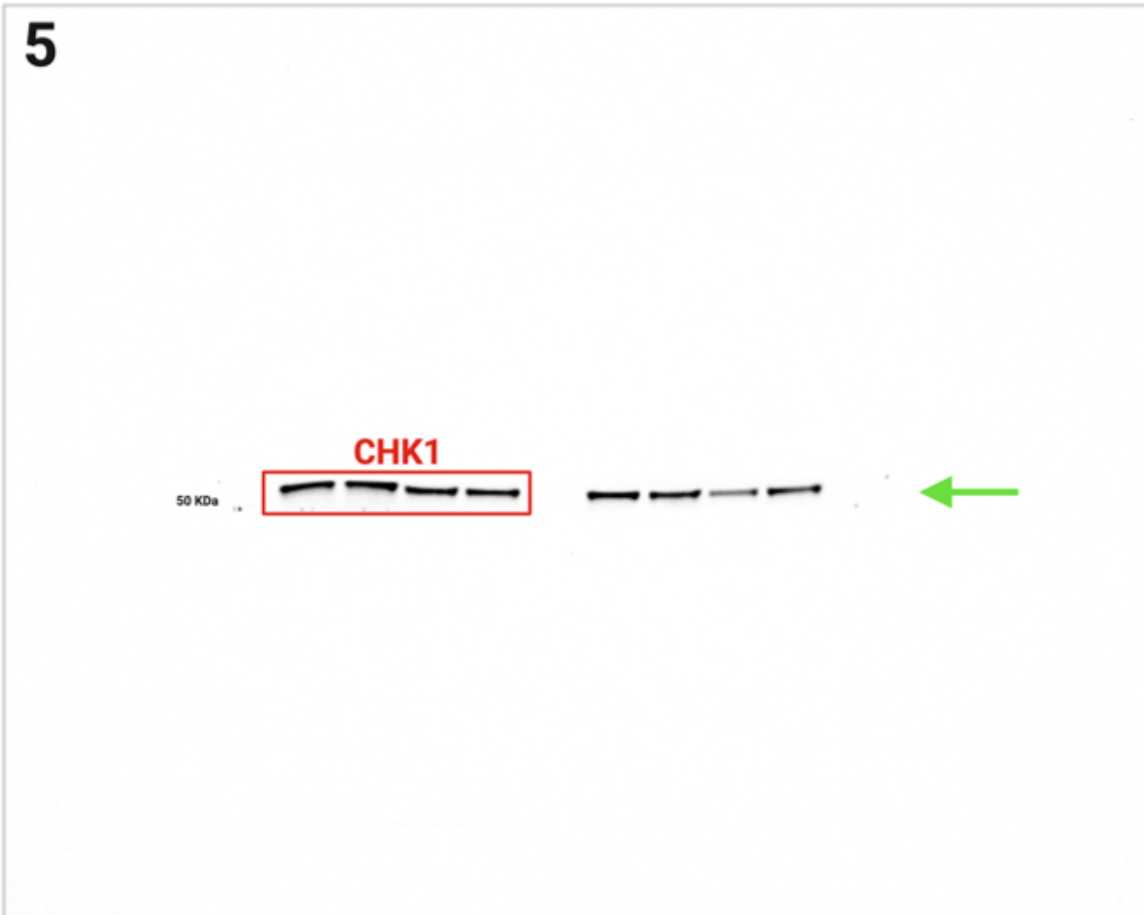
5
CHK1

Supplemental material 1: Original western blots pictures utilized to assemble figure 3b. Molecular weights are indicated. Pictures 1 to 4 show different exposure times of the same filter (cut for incubation with different antibodies). Picture 5 shows the exposure of the filter part indicated by the green arrow after stripping and re-incubation with a different antibody. The red squares indicate the images parts used to assemble figure 3b with the corresponding protein indicated above.

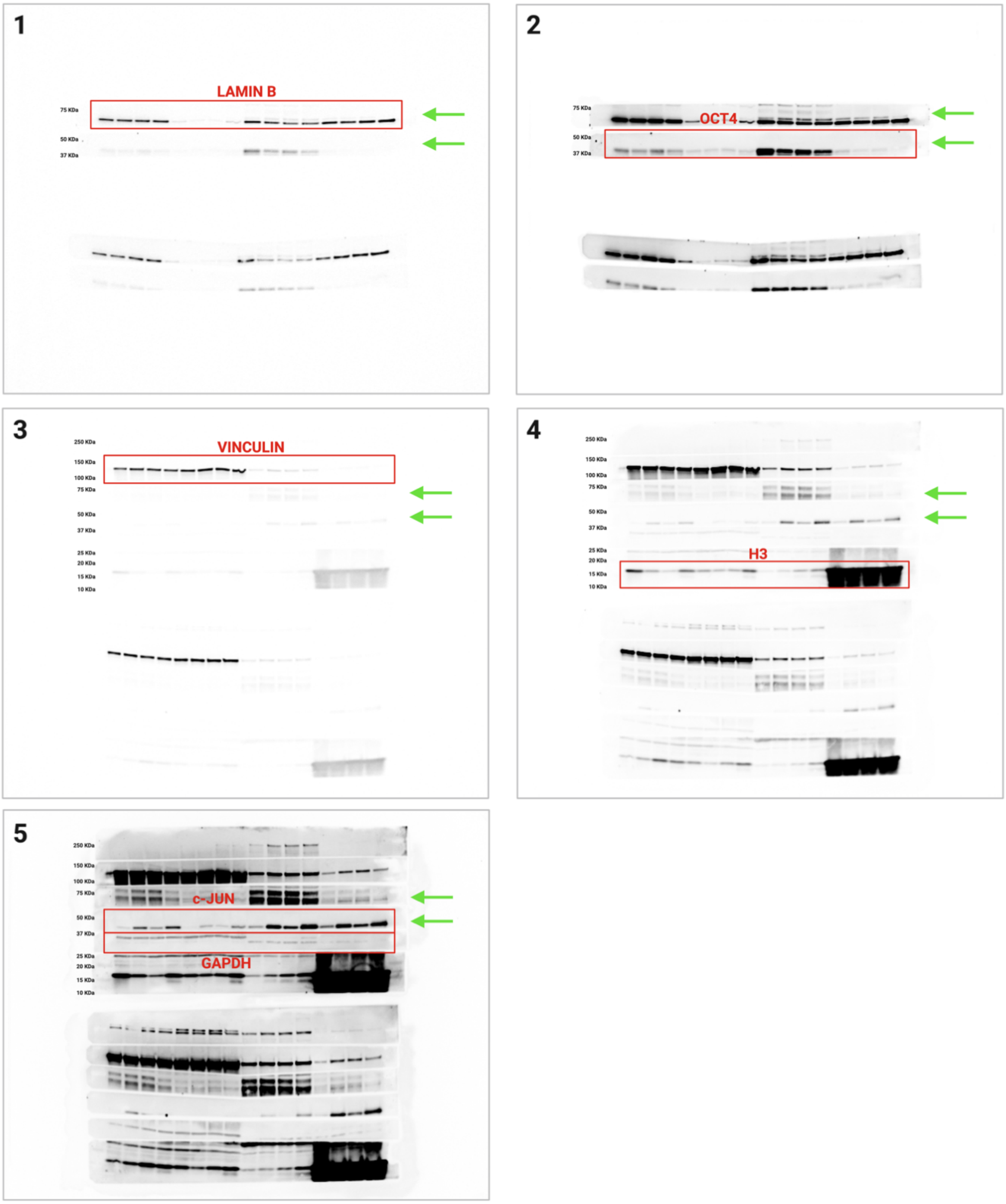
1
LAMIN B
75 KDa
50 KDa
37 KDa
2
OCT4
75 KDa
50 KDa
37 KDa
3
VINCULIN
250 KDa
150 KDa
100 KDa
75 KDa
50 KDa
37 KDa
25 KDa
20 KDa
15 KDa
10 KDa
4
H3
250 KDa
150 KDa
100 KDa
75 KDa
50 KDa
37 KDa
25 KDa
20 KDa
15 KDa
10 KDa
5
c-JUN
GAPDH
250 KDa
150 KDa
100 KDa
75 KDa
50 KDa
37 KDa
25 KDa
20 KDa
15 KDa
10 KDa

Supplemental material 2: Original western blots pictures utilized to assemble figure 5f. Molecular weights are indicated. Pictures 3 to 5 show different exposure times of the same filter (cut for incubation with different antibodies). Pictures 1 and 2 show the exposure of the filter parts indicated by the green arrows after stripping and re-incubation with a different antibodies. The red squares indicate the images parts used to assemble figure 5f with the corresponding protein indicated above.

1

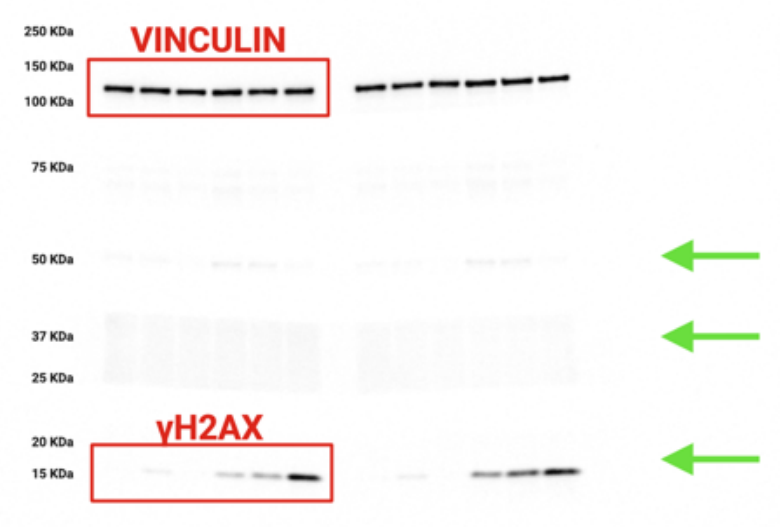
250 KDa
150 KDa
100 KDa
75 KDa
50 KDa
37 KDa
25 KDa
20 KDa
15 KDa
VINCULIN
γH2AX

2

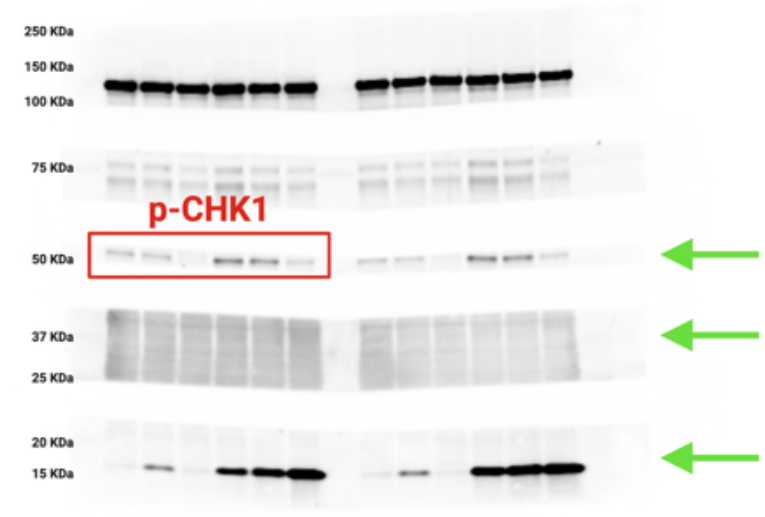
250 KDa
150 KDa
100 KDa
75 KDa
50 KDa
37 KDa
25 KDa
20 KDa
15 KDa
p-CHK1

3

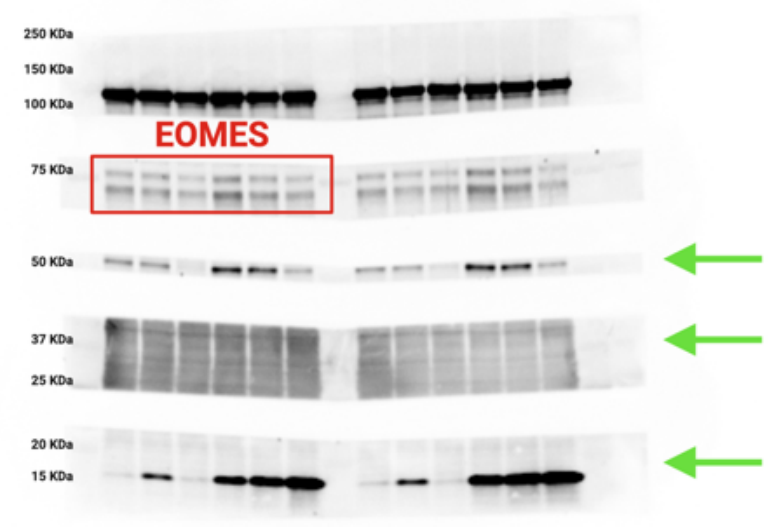
250 KDa
150 KDa
100 KDa
75 KDa
50 KDa
37 KDa
25 KDa
20 KDa
15 KDa
EOMES

4

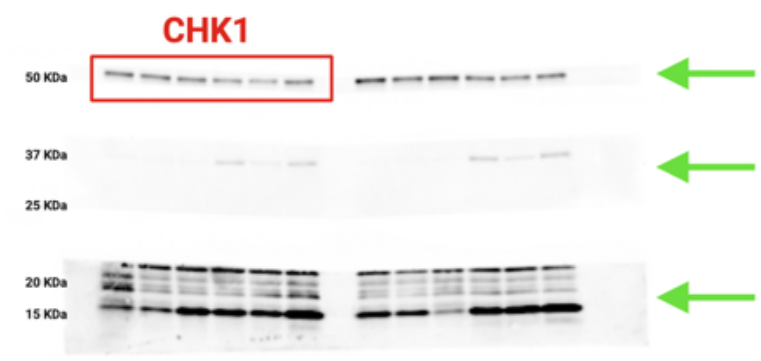
50 KDa
37 KDa
25 KDa
20 KDa
15 KDa
CHK1

5

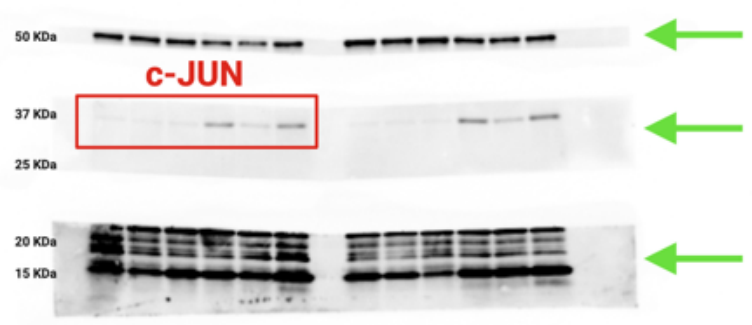
50 KDa
37 KDa
25 KDa
20 KDa
15 KDa
c-JUN

6

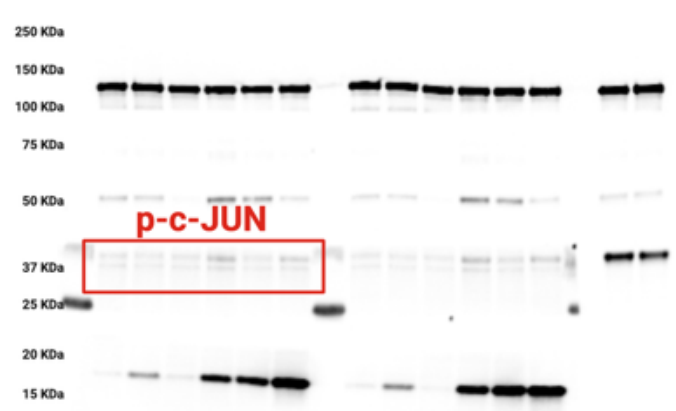
250 KDa
150 KDa
100 KDa
75 KDa
50 KDa
37 KDa
25 KDa
20 KDa
15 KDa
p-c-JUN

Supplemental material 3: Original western blots pictures utilized to assemble figure 6c. Molecular weights are indicated. Pictures 1 to 3 show different exposure times of the same filter (cut for incubation with different antibodies). Pictures 4 and 5 show the exposure of the filter parts indicated by the green arrows after stripping and re-incubation with a different antibodies. Picture 6 shows the same samples loaded on an additional filter. The red squares indicate the images parts used to assemble figure 6c with the corresponding protein indicated above.
